# The Complex Interplay between Evolutionary Flexibility and Diversification in a Family of Spiders

**DOI:** 10.1093/sysbio/syaf070

**Published:** 2025-10-13

**Authors:** Guanliang Meng, Lars Podsiadlowski, Dimitar Dimitrov, Bernhard A Huber

**Affiliations:** Zoological Research Museum Alexander Koenig, LIB, Adenauerallee 127, 53113, Bonn, Germany; Zoological Research Museum Alexander Koenig, LIB, Adenauerallee 127, 53113, Bonn, Germany; University Museum of Bergen, University of Bergen, P.O. Box 7800, 5020, Bergen, Norway; Zoological Research Museum Alexander Koenig, LIB, Adenauerallee 127, 53113, Bonn, Germany

**Keywords:** Allometry, diversity, Pholcidae, phylogenomics, speciation, trait evolution

## Abstract

Understanding the mechanisms of how morphological traits drive speciation and contribute to species richness is pivotal in evolutionary biology. In this context, the evolutionary flexibility of morphological traits may play a significant role. Using the diverse daddy long-legs spiders, Pholcidae, which currently includes some 2000 described species, we explored the interplay between speciation rate, trait evolution rate, microhabitat shift rate, species richness, interspecific variability of body size, leg length, relative leg length, and leg proportions. We applied a combination of large-scale genomic and taxonomic sampling, and phylogenetic and comparative analyses to assess the dynamics of diversification and evolutionary flexibility (measured as either the standard variance or disparity of traits), as well as their interactions. We found that increased evolutionary flexibility is accompanied with accelerated rates in speciation and trait evolution, and with higher species richness. We also observed near-isometry of leg length and body size in the species-rich lineages, suggesting stabilizing selection, whereas positive allometry in the species-poor lineages indicates directional selection. Additionally, we found a positive correlation between trait evolution rates and microhabitat shifts. We argue that environmental heterogeneity and frequent microhabitat shifts may contribute to the origin and maintenance of evolutionary flexibility, which in turn influences the organisms’ ability to exploit new resources and habitats. On the other hand, our study suggests that a lack of evolutionary flexibility (e.g., due to dwarfism or gigantism) could be an evolutionary dead end. This study enhances our understanding of the mechanisms of diversification, demonstrating that evolutionary flexibility of morphological traits may vary among closely related taxa and is likely to have contributed to the uneven distribution of biodiversity across the tree of life.

The mechanisms underlying the uneven distribution of biodiversity among clades represent one of the core questions in evolutionary biology. Organisms with higher levels of evolutionary flexibility are able to adapt to different and changing environments more effectively, which may contribute to their diversification (Ricklefs and Renner 1994; Pfennig et al. 2010; Wright and Turko 2016; Aiello et al. 2021; Mowery et al. 2022). On the other hand, taxa exhibiting evolutionary stasis experience slow rates of morphological evolution and have lower species diversity (Brownstein et al. 2024). Although some studies suggest that speciation rates can be decoupled from phenotypic evolution (e.g., Rabosky 2013; Crouch and Ricklefs 2019), others have found a correlation between speciation rate and the evolution of phenotypic traits (e.g., Cardillo et al. 2005; Mahler et al. 2010; Etienne et al. 2012; Cooney and Thomas 2021; Cerezer et al. 2023). However, whether this is a causal relationship remains to be determined—taxonomic diversification can follow, coincide with, or even precede increases in morphological disparity (Jablonski 2017; Folk et al. 2019). Many authors have tried to identify key innovations that might have played important roles in the radiation of different groups by offering novel ecological opportunities (e.g., Lee et al. 2014; Gillet et al. 2019; Siqueira et al. 2020), or have focused on the correlation between trait values and speciation/extinction rates (Cardillo et al. 2005; Etienne et al. 2012). Meanwhile, recent development of sophisticated models (e.g., Venditti et al. 2011; Rabosky 2014) allows to assess the relationship between the rates of trait evolution and speciation more precisely (Rabosky et al. 2013; Beltran et al. 2021; Cooney and Thomas 2021; Cerezer et al. 2023). Few studies have focused on invertebrates (e.g., arthropods), which include more than 50% of all known animals (Scheffers et al. 2012).

Body size has fundamental impacts on the way species interact with the environment (Arnold et al. 2017; Shao et al. 2023), affecting their fitness, ecology, physiology, and life history (LaBarbera 1989; Smith and Belk 2018). Previous studies show that larger body size can lead to higher fitness by a range of mechanisms, like better escape from predators, higher mating success, and higher dispersal ability (Marshall and Gittleman 1994; Kingsolver and Pfennig 2004; Hone and Benton 2005; Avaria-Llautureo et al. 2021). Large body size can result from both neutral and selective processes. In a neutral process, the increase in body size arises from the increase of trait variance over time following a Brownian motion model, without the influence of directional selection (Harvey and Pagel 1991). On the other hand, Cope’s rule describes an active process, hypothesizing that body size tends to increase over the course of evolution (Stanley 1973), and it has been suggested that competition, climatic cooling, and selection are among the drivers (Kingsolver and Pfennig 2004; Hunt and Roy 2006; Benson et al. 2014). Cope’s rule has received mixed support and remains controversial. Evidence supporting the rule includes studies by Baker et al. (2015), Benson et al. (2014), and Heim et al. (2015). Evidence against the rule has been presented by Jablonski (1997), Dale Guthrie (2003), Gotanda et al. (2015), Knouft and Page (2003), Monroe and Bokma (2010), Sallan and Galimberti (2015), and Waller and Svensson (2017). On the other side, the size-grain hypothesis (SGH) (Kaspari and Weiser 1999) suggests that, as body size decreases, organisms perceive the Earth's surface as more rugged compared with larger organisms. The relatively increased complexity may provide opportunities for further specialization.

Legs or appendages in animals are mostly responsible for adhesion and locomotion (Holmes et al. 2006; Labonte and Federle 2013; van Griethuijsen and Trimmer 2014; Silva et al. 2021). Absolute and relative leg lengths have important implications. Leg length controls stride length, affecting stride frequency and walking speed (Taylor et al. 1980; Kaspari and Weiser 1999). The SGH suggests that smaller body size comes with shorter legs, because they facilitate access to small interstices that can provide food and shelter (Kaspari and Weiser 1999). Therefore, leg length and relative leg length affect habitat selection (Kaspari and Weiser 1999; Li et al. 2011). Similar to Cope’s rule, the SGH is supported by some studies (e.g., Kaspari and Weiser 2007; Pearce-Duvet et al. 2011) but not by others (e.g., Parr et al. 2003; Teuscher et al. 2009). Like body size, leg length is also correlated with locomotion cost (Pontzer 2007; Grossi et al. 2016) and dispersal ability (Phillips et al. 2006; Corcobado et al. 2012). Terrestrial animals with smaller body sizes and shorter legs are found to use more energy (relative to body mass) than larger animals when traveling the same distance (Pontzer 2007). Leg length is indirectly linked with speciation via dispersal ability (Cadotte 2006; Venail et al. 2008; Claramunt et al. 2012; Czekanski-Moir and Rundell 2019).

Traits are often correlated both developmentally and genetically (a phenomenon known as integration, as opposed to modularity) (Armbruster et al. 2014; Conner et al. 2014; Evans et al. 2021) and so are their evolutionary flexibilities. The relationship between the measure of a trait (e.g., leg length) and another reference trait (e.g., body size) is called morphological scaling or allometric scaling, and can be formulated as: log *y* = log *b* + *a* log *x*, where *y* and *x* are the values for the two traits, respectively; *b* is the intercept and *a* is the allometric scaling factor. Such allometric scaling relationships are frequently observed in morphological, physiological, and life-history traits (e.g., West et al. 1997; Andresen et al. 2002; Bolstad et al. 2015; White et al. 2019; Chong et al. 2024). Allometric relationships between traits may be ontogenetic, static, or evolutionary, depending on whether the relationship occurs among different developmental stages of the same individual, different individuals at the same developmental stage within the same population, or across different species (Cock 1966; Gould 1966; Cheverud 1982). Understanding whether morphological scaling parameters are under evolutionary constraints, and the nature of such constraints, may provide insights into the formation of phenotypic and species diversity. On the one hand, the allometric-constraint hypothesis proposes that factors such as functional and physiological mechanisms strongly restrict the evolution of morphological scaling parameters and the evolution of related morphological traits (Gould 1966; Pélabon et al. 2014; Voje et al. 2014; Voje et al. 2022). On the other hand, if variations in genetics and developmental processes that regulate the growth of related traits exist, natural selection may act on these variations, potentially driving the evolution of allometry—unless strong negative selection or stabilizing selection prevents such changes (Houle et al. 2019). Artificial selection experiments have shown that natural selection could drive the evolution of the slope and intercept of scaling relationships, and pleiotropy is thought to be responsible for the rapid reversal to the initial slope once selection is suspended (Frankino et al. 2005; Bolstad et al. 2015; Houle et al. 2019). The slope is found to evolve slower than the intercept (Egset et al. 2012; Pélabon et al. 2014; Voje et al. 2014; Bolstad et al. 2015; Voje et al. 2022), suggesting the slope is subject to stronger evolutionary constraints. Additionally, natural selection may also break trait–trait correlations, resulting in phenotypic modularity and high variability in each trait (Voje et al. 2022). However, it is unclear how morphological scaling is related to phenotypic divergence and species diversification. For example, are there any differences in the allometric parameters (i.e., slope and intercept) between species-rich and species-poor sister clades? Are allometric parameters in different parameter spaces under the same direction and strength of natural selection, and how does this correlate with evolutionary flexibility?

Spiders (Araneae), with their diverse range of body sizes and leg lengths (Wolff et al. 2022; Shao et al. 2023), make an ideal group for studying the evolution of these traits. Spider body size and leg length variation have been attributed to various factors and shown to affect spider biology. Larger spiders tend to win intraspecific male–male fights (Wells 1988) and run faster (Foellmer et al. 2011). Larger-bodied spiders typically undergo more molts and longer intermolt intervals to reach sexual maturity compared with smaller-bodied spiders (Head 1995), and the size of a spider also significantly influences the diversification of both web architectures and reproductive strategies (Sensenig et al. 2011; Dong et al. 2023). Relative leg length varies a lot among spiders and has different implications. Some spiders (e.g., Salticidae) have very short relative leg lengths, whereas others (e.g., Agelenidae and Pholcidae) generally have very long legs (Pechmann et al. 2010). Spiders that live in silk-lined burrows tend to have larger bodies with relatively shorter legs. Conversely, spiders that build aerial webs generally have smaller bodies and relatively longer legs. Spiders that hunt exhibit an intermediate body size and morphology (Shao et al. 2023). We hypothesize that the evolutionary flexibility of morphological traits—measured by trait disparity and/or variance—rather than their absolute size, is what truly matters and may have contributed to species diversification. Indeed, fossil data analyzed by Solórzano et al. (2020) suggest that reduced body mass disparity among crocodilians is linked to a higher risk of extinction. We explore this topic more thoroughly by using spiders as a model system.

Diversification in spiders has previously been attributed to: (1) silk innovations and web design/loss (Bond and Opell 1998); (2) the evolution of venom (King and Hardy 2013); (3) the evolution of adhesive setae (Wolff et al. 2013); (4) the transformation of the second pair of book lungs into tracheae (Schmitz 2016); (5) the coevolution with insect prey (Liu et al. 2016); (6) geological processes and climate changes (Xu et al. 2015); (7) sexual selection (Gage et al. 2002); and (8) microhabitat shifts (Eberle et al. 2018). The impact of body size and leg length evolution, as well as their allometric pattern on diversification in spiders, have rarely been studied (Moya-Laraño et al. 2008; Kuntner et al. 2019; Kuntner and Coddington 2020; Wolff et al. 2022; Shao et al. 2023). Here, we investigate the relationships between evolutionary flexibility, diversification, and allometry using the daddy long-legs spiders Pholcidae as a study system. Pholcidae is one of the largest and ecologically most diverse spider families, including slightly over 2000 described species (World Spider Catalog 2025). The family has a worldwide distribution, predominantly in tropical and subtropical regions (Huber et al. 2018). Pholcids occupy a wide range of microhabitats, which can be classified into three main types: “space,” “leaf,” and “ground” (Eberle et al. 2018). They are highly variable in body size, shape, and color; and, despite their vernacular name, include both species with very short legs (1–2 mm) and species with leg spans of up to 180 mm.

## Materials and Methods

### Taxonomic Sampling and Target Capture Sequencing

For the phylogenomic analyses, we selected 96 pholcid species (Supplementary Table S1; see https://doi.org/10.5061/dryad.r2280gbnh for details) representing all five formally recognized subfamilies (Arteminae, Smeringopinae, Pholcinae, Modisiminae, and Ninetinae) (Huber 2011), with a strong emphasis on those groups with unclear or dubious placement in the latest and largest molecular phylogeny of the family (Eberle et al. 2018). This concerned Ninetinae, *Artema* and *Priscula*, which were identified as the three “most obvious gaps” in pholcid phylogeny (Huber et al. 2018). The sister-group relationships Pholcinae + Smeringopinae, and Modisiminae + “other Arteminae” (i.e., the traditional Arteminae without *Artema*) appear beyond doubt (Eberle et al. 2018; Huber et al. 2018); therefore, we have a weak representation of these four groups. We use an operational division of Pholcidae into seven subfamily-level groups: Pholcinae, Smeringopinae, Modisiminae, Ninetinae, *Artema*, “other Arteminae,” and *Priscula*. For the sake of simplicity, we will use “subfamilies” as a shorthand for “subfamily-level groups” throughout the manuscript; we use “Arteminae” as a shorthand for “other Arteminae,” and “traditional Arteminae” to address the original concept (*Artema* + “other Arteminae”). Vouchers of all analyzed species are deposited in ZFMK (Zoologisches Forschungsmuseum Alexander Koenig, Bonn, Germany). Additionally, we chose the four families most closely related to Pholcidae as outgroups, following the topology of Kulkarni et al. (2021), that is, *Scytodes* sp. (Scytodidae), *Maoriata* sp. (Orsolobidae), *Hypochilus gertschi* (Hypochilidae), and *Loxosceles reclusa* (Sicariidae), downloaded from NCBI. They were analyzed together with our 96 newly sequenced samples.

DNA was extracted with the DNeasy Blood & Tissue Kit (Qiagen, Hilden, Germany) following the manufacturer’s instruction. Hybrid enrichment followed the approach described in Sann et al. (2018). Indexed gDNA libraries for Illumina sequencing were constructed using the SureSelect XT HS2 Library Preparation Kit (Cat # 5500-0147, Lot # 0006583499, Agilent Technologies, USA), which were then hybridized to the spider‐specific probe set for ultra-conserved elements (UCE) (Kulkarni et al. 2020) and amplified using the SureSelect XT HS2 Target Enrichment Kit (Cat # 5191-6690, Lot # 0006599172, Agilent Technologies, USA) following the manufacturer’s instruction. Sequencing of captured gDNA libraries was performed by Macrogen Europe (Amsterdam, NL) using pair-end (PE) 150-bp strategy. Each specimen was sequenced with ca. 1.5 Gb of data. More details on the experiment are available in the Supplementary Text.

### The UCE Matrices

To study the subfamily-level phylogenetic relationships of Pholcidae, we constructed 10 different matrices based on the UCE data (see Supplementary Fig. S1a).

#### Data quality control and sequence assembly

The raw data of all 100 samples except *L. reclusa* were filtered with fastp (v0.23.1) (Chen et al. 2018). Reads were filtered out if: (1) one read contained more than 10 Ns; (2) more than 40% of bases were unqualified; (3) reads were duplicates; and (4) reads were shorter than 40 bp. Clean reads were assembled into contigs using SPAdes (v3.14.1) (Prjibelski et al. 2020) in Phyluce (v1.7.1) (Faircloth 2016). To detect potential cross-contaminations among species, “all-blast-all” analysis of the assembled contigs was performed with megablast in Blast+ (v2.9.0) (Camacho et al. 2009) under the default parameters. The blast hits with similarity ≥99% and alignment length ≥100 bp were kept and further checked for biological sources by blasting their sequences against a local NT database. We used the following criteria to keep or remove sequences: if the abundance difference (output by SPAdes) between the query and subject sequences was less than 10X, both sequences were marked as “removed.” If both sequences had abundances ≥20X and their abundance difference exceeded 10X, the sequences with lower abundance was marked as “removed.” Any sequences marked as “removed” were excluded from the assembly.

#### Multiple sequence alignment

The UCE sequences of all samples were extracted with Phyluce, aligned with MAFFT (v7.475) (Katoh and Standley 2013) and trimmed with Gblocks (v0.91b) (Castresana 2000) (both implemented in Phyluce) and TrimAl (v1.4.rev15) (Capella-Gutierrez et al. 2009) (-automated 1).

Six data matrices (1–6, see Supplementary Fig. S1a) were generated based on different taxonomic occupancy (40%, 50%, 60%, 70%, and 80%) and locus length (≥50 bp vs. ≥150 bp). Another four data matrices (matrices 7–10) were generated based on matrix 3 to test if there are systematic biases in our data (see the *Systematic Bias Assessment* section and Supplementary Fig. S1a).

### The Six-Gene Matrices

To better understand the evolutionary dynamics of pholcids and to assess the impact of trait evolution on pholcid diversification, we constructed the most comprehensive pholcid phylogeny so far, using six genes (*12S rDNA*/*16S rDNA*/*18S rDNA*/*28S rDNA*/*Histone 3* [*H3*]/*Cytochrome c oxidase I* [*CO1*]) combining newly generated and public data from the following sources: (1) the current UCE data; (2) 101 newly sequenced specimens (84 *CO1*, 2 *H3*, 1 *12S*, 3 *18S*, 11 *28S*) following Eberle et al. (2018); (3) the data set of Eberle et al. (2018) (almost 600 species); and (4) all other pholcid sequences available in GenBank (access date: 26 October 2022). In total, we were able to obtain data for 991 species (including 35 outgroup species) and 424 *12S*, 521 *16S*, 462 *18S*, 450 *28S*, 426 *H3*, 873 *CO1* sequences (Supplementary Table S2).

#### Screening for the 12S/16S/18S/28S/H3/CO1 sequences

The *18S* and *28S* genes in the SPAdes assemblies were identified with RNAmmer (v1.2) (Lagesen et al. 2007), which uses hidden Markov models trained on rRNA data for different super-kingdoms (Archaea, Bacteria, and Eukaryotes). The deep-learning-based software, Tiara (v1.0.2) (Karlicki et al. 2021), was employed to screen for mitochondrial sequences (--to_fasta mit pla pro --prob_cutoff 0.65 0.60 --probabilities --min_len 500 -t 8). The mitochondrial sequences were annotated by MitoZ (v3.4) (Meng et al. 2019) for *12S* and *16S* genes. A method combining tBlastn (v2.2.26) (Camacho et al. 2009) and Genewise (v2.4.1) (Birney et al. 2004) is implemented in MitoZ and was used to search for *H3* and *CO1* genes. In all cases, candidate gene sequences not overlapping with the data set of Eberle et al. (2018) were identified with Blast+ and filtered out. Next, to exclude potential false positives, the remaining sequences were aligned with the corresponding genes of Eberle et al. (2018) using MAFFT (v7.487) with the mafft-linsi algorithm (for *CO1* and *H3*) or the mafft-xinsi algorithm (for rDNA genes). The alignments were checked by eye in AliView (v1.28) (Larsson 2014). Whenever there were multiple copies of the same gene for one sample, only the candidate copies with the highest abundances (from SPAdes) were kept, because copies with lower abundances were assumed to be artifacts (e.g., assembly errors, and contaminations). Finally, all retained sequences were blasted against the NT database to further confirm their biological sources.

#### Multiple sequence alignment

For the protein-coding genes *CO1* and *H3*, DNA sequences were translated into protein using BioPython (v1.78) (Cock et al. 2009). Next, protein multiple sequence alignments (MSAs) were constructed using the mafft-linsi algorithm of MAFFT (v7.487), which then assisted the construction of nucleotide-level MSAs with pal2nal.pl (Suyama et al. 2006). This helps avoid the introduction of biologically meaningless frameshifts to the alignments (Suyama et al. 2006). The alignments of rDNA genes (*12S, 16S, 18S*, and *28S*) were constructed based on secondary structure information using the mafft-xinsi algorithm in MAFFT (v7.487) and MXSCARNA (Tabei et al. 2008). Poorly aligned regions in the MSAs were trimmed with Gblocks (-b5 = h), TrimAl (-automated 1), and ClipKIT (v1.1.3) (Steenwyk et al. 2020). For the ClipKIT program, we tested eight different trimming strategies (--modes gappy, kpi, kpic, kpic-gappy, kpic-smart-gap, kpi-gappy, kpi-smart-gap, and smart-gap). Thus, we obtained 10 six-gene matrices in total.

### Systematic Bias Assessment

#### Information content and substitutional saturation assessment

To detect systematic biases in our UCE data sets, the trimmed MSAs were evaluated for their phylogenetic information. For each locus, we calculated its average GC content, the interquartile range of GC content, the number and proportion of missing data (i.e., gaps and Ns), the number and proportion of parsimonious informative sites, the length of the MSA, the taxon number of the MSA, and a saturation plot using custom scripts. The density plots of these features as well as the missing data distribution plot were created using ggplot2 (v3.3.6) (Ginestet 2011) and R (v4.2.0) (R Core Team 2022). Substitutional saturation was also assessed by PhyloMAd (v1.2) (Duchene et al. 2022) based on measures of entropy. Loci with high risks of saturation were excluded from matrix 3 and thus matrix 7 was created (Supplementary Fig. S1a).

#### Test of stationarity, homogeneity, and treelikeness

Many evolutionary models assume stationarity (constant nucleotide/amino acid frequencies over time), reversibility (substitution rates in both directions are equal), homogeneity (constant substitution rates over time), and treelikeness (different sites of an MSA share the same evolutionary history). IQ-TREE (v2.2.0) (Minh et al. 2020) was used to test the assumptions of stationarity and homogeneity (--symtest-only). The two assumptions were rejected when *P*-values (SymPval) < 0.01 were found. The likelihood mapping method (Strimmer and von Haeseler 1997) in IQ-TREE was used and 100,000 random quartets of taxa were drawn from the concatenated MSA of all loci and visualized in a single triangular graph. The more quartets cluster at the three corners of the triangular graph, the more suitable the data are to construct the phylogeny (Strimmer and von Haeseler 1997).

#### Test of base compositional heterogeneity

We calculated the compositional heterogeneity (relative composition frequency variability, RCFV) with BaCoCa (v1.109) (Kück and Struck 2014). RCFV is a measurement of absolute deviation from the mean nucleotide (or amino acid) frequency in the data set. The higher the RCFV values, the higher the compositional heterogeneity, which can be seen in fast-evolving clades and may have negative impact on phylogenetic inference (Cai et al. 2022). Compositional homogeneity was also checked with IQ-TREE using the χ^2^ test, in which a sequence is labeled as “failed” if its character composition differs significantly (*P*-value < 0.05) from the average composition of the alignment. To alleviate the impact of compositional heterogeneity on phylogenetic tree construction, we created an RY-coding data set (matrix 8) from matrix 3 using the script at https://raw.githubusercontent.com/ballesterus/Utensils/master/RYplace.py. Additionally, we selected compositionally homogenous sites from the concatenated MSA of matrix 3 and created matrix 9 using the BMGE program (v1.12) (Criscuolo and Gribaldo 2010). Using the same method, we also created matrix 10 from matrix 7 (Supplementary Fig. S1a).

#### Intra-locus recombination detection

Coalescence-based phylogenetic tree construction methods generally assume there is no recombination within each locus, whereas different loci are in linkage equilibrium (Zhu et al. 2022). We employed the program 3SEQ (v1.8.0) (Lam et al. 2018) to detect the significant presence of intra-locus recombination (*P*-value < 0.05) using a non-parametric test for mosaicism (Boni et al. 2007). Before the analysis, a 3000 * 3000 * 3000 *P*-value table was pre-computed.

### Phylogenetic Inference Based on the UCE Matrices

To build a robust phylogenetic hypothesis, we used both concatenation- and coalescence-based methods using IQ-TREE (v2.2.0), RAxML-NG (v1.1) (Kozlov et al. 2019), ASTRAL (v5.7.8) (Zhang et al. 2018), SVDquartets (Chifman and Kubatko 2014) implemented in PAUP∗ (v4a168) (Swofford 2002), and PhyloBayes-MPI (v1.8) (Lartillot et al. 2013) (Supplementary Fig. S1b).

#### Maximum likelihood and Bayesian inference

We applied IQ-TREE to all data matrices except matrices 9 and 10 using a partitioned strategy (-p). For matrices 9 and 10, we applied the unpartitioned strategy due to their short lengths (8.6 Kbp). Best-fit models of molecular evolution were determined by ModelFinder (Kalyaanamoorthy et al. 2017) in IQ-TREE automatically (-m MFP + MERGE). To overcome local optima during heuristics, we performed 10 independent runs on each data set, with a smaller perturbation strength (-pers 0.2) and a larger number of stop iterations (-nstop 500) (Nguyen et al. 2015; Liu et al. 2024). Branch supports were evaluated with 2000 ultrafast bootstrap (UFBoot) (Minh et al. 2013) considering potential model violations (-B 2000 -bnni --sampling GENESITE). The SH-aLRT branch test (Guindon et al. 2010) was performed using 2000 bootstrap replicates.

For comparison, we also used both RAxML-NG (two runs) and PhyloBayes-MPI with matrix 3, which balances the number of loci and taxon coverage. Compared with IQ-TREE, RAxML-NG is less sensitive to different starting trees (Liu et al. 2024). During each RAxML-NG run, the GTR+R4+I+FO model, and 25 parsimony starting trees + 25 random starting trees were used, and the number of bootstrap replicates was automatically determined (--bs-trees autoMRE) (Pattengale et al. 2009). The infinite mixture model, CAT-GTR+G4 model (Lartillot and Philippe 2004), was used in our PhyloBayes-MPI analysis. It can model both rate-heterogeneity across sites and pattern-heterogeneity across sites (i.e., across-site compositional heterogeneity), and is less sensitive to systematic errors than classical empirical models (Lartillot et al. 2013). It is increasingly used to resolve phylogenetic controversies despite of its intensive computational demands (e.g., Ballesteros et al. 2022; Cai et al. 2022). Two independent PhyloBayes-MPI runs were performed, and convergences of the continuous parameters of the model and the tree spaces were evaluated using the tracecomp and bpcomp commands in PhyloBayes-MPI. One-third of samplings of each run was used as burn-in, and a majority consensus tree was generated by combining the trees of the two runs with the bpcomp command.

#### Coalescence-based inference

We used ASTRAL and SVDquartets with matrix 3. In the ASTRAL analysis, individual gene trees were firstly estimated by IQ-TREE as described above. To reduce noise, internal nodes of gene trees with low branch supports (≤30) were collapsed (Zhang et al. 2018) and ASTRAL was run with default parameters. ASTRAL is sensitive to gene tree estimation errors (Mirarab et al. 2021); therefore, we also used SVDquartets, bypassing the gene tree construction step. In the SVDquartets analysis, we evaluated all quartets (evalQuartets = all) under the multispecies coalescent model (treemodel = mscoalescent), which allows for gene tree discordance due to incomplete lineage sorting. The “Erik+2” normalization was used (plus2 = yes) to consider the heterogeneity among sites and lineages (Fernandez-Sanchez and Casanellas 2016). The QFM algorithm (Reaz et al. 2014) was used to search for an optimal tree under the maximum quartet compatibility algorithm (treeInf = QFM), and 200 multilocus bootstrap replicates were sampled (bootstrap = multilocus nreps = 200).

#### Constrained tree search and topology test

Using matrix 3 and the same parameter settings of IQ-TREE as described above, we performed two constrained tree searches. Based on previous studies (Bruvo-Mađarić et al. 2005; Huber 2011; Dimitrov et al. 2013; Eberle et al. 2018; Huber et al. 2018), we tested two hypotheses: (1) the traditional Arteminae (i.e., “other Arteminae” + *Artema*) is a monophyletic group. (2) Ninetinae is sister to all other pholcids.

Finally, topology tests with the bootstrap proportion using the RELL method (bp-RELL) (Kishino et al. 1990) and Expected Likelihood Weight (c-ELW) (Strimmer and Rambaut 2002) were performed across all unconstrained and constrained trees using IQ-TREE. DiscoVista (Sayyari et al. 2018) was used to visualize the relative gene frequency support for different topologies, whereas the likelihood mapping method (Strimmer and von Haeseler 1997) in IQ-TREE was applied to visualize the site support. All trees were visualized with iTOL (v6.5.8; https://itol.embl.de/) (Letunic and Bork 2021).

#### Divergence time calibration

To date the tree, we used eight calibration points based on fossil data from the PaleoDB database (https://paleobiodb.org) and publications (Wunderlich 1988; Penney 2006; Serrano-Sánchez et al. 2015; García-Villafuerte and Valdez-Mondragón 2020; Magalhaes et al. 2020) (Supplementary Table S3). We followed Magalhaes et al. (2020) for the justifications of the fossil assignments; see Supplementary Table S3. Briefly, the oldest representative fossil of each clade was used to calibrate its divergence from the sister group (stem-dating), following Benton and Donoghue (2006) and Magalhaes et al. (2020). The soft-bound strategy was employed. We set the age bounds of the root (Synspermiata + Hypochilidae) as follows: the minimum bound (164 million years ago, Ma) reflects the placement of the genus *Eoplectreurys* as a stem-Synspermiata, based on the oldest known fossil species, *E. gertschi* Selden & Huang (Gao and Ren 2006; Magalhaes et al. 2020); the conservative maximum bound (400 Ma) was chosen because no Araneae stem fossils older than 383 Ma are known (Magalhaes et al. 2020). The MCMCTREE program in PAML (v4.10.6) (Yang 2007) and node-dating strategy were applied to calibrate the divergence times based on fossil records following the recommendations in Parham et al. (2012). Five independent MCMCTree runs were performed and the convergence of MCMC (Markov chain Monte Carlo) chains was checked.

### Phylogenetic Inference Based on the Six-Gene Matrices

For each of the 10 six-gene matrices, a constrained tree search analysis was conducted with IQ-TREE using the partitioned strategy and the same parameter settings as in the UCE-tree analysis. As a constraint we used the tree from the IQ-TREE partitioned maximum likelihood (ML) analysis of matrix 3. To explicitly account for the impact of topological uncertainty on our results while ensuring analytical tractability, we selected three representative trees for subsequent comparative analyses: a primary tree based on the ClipKIT smart-gap trimmed-MSA; and two alternative trees based on the ClipKIT gappy and kpic-smart-gap trimmed-MSAs. These trees were chosen based on two criteria: minimum number of species obviously misplaced at subfamily level; and monophyly of uncontested genera. For each of the three trees (hereafter “target trees”), divergence times were estimated using TreePL (v1.0) (Smith and O’Meara 2012) and the R function congruify.phylo() in geiger (v2.0.11) (Pennell et al. 2014), with our dated UCE-matrix 3 tree (see the *Divergence time calibration* section) used as the reference tree in all analyses. Because our reference tree is a subset of each of the target trees in terms of tip taxa and topology, the concordant nodes between the reference tree and a target tree can be easily determined by the congruify.phylo() function (Eastman et al. 2013). Thus, the divergence times of all nodes in the reference tree serve as calibration points for the target tree, which were passed to TreePL via congruify.phylo() to calibrate the node ages of the target tree (Eastman et al. 2013).

Finally, to further assess the robustness of our phylogenetic inference, we conducted two sets of unconstrained analyses: (1) unpartitioned IQ-TREE analyses combing all UCEs in matrix 3 with each of the three six-gene matrices (smart-gap, gappy, and kpic-smart-gap), respectively, using best-fitting models selected by ModelFinder (Kalyaanamoorthy et al. 2017); and (2) partitioned RAxML-NG analyses that combined the 55 UCE loci with the highest numbers of parsimony-informative sites in matrix 3 with each of the three six-gene matrices, respectively, under the GTR+R4+ I+FO model.

### Comparative Analyses

We focus on four traits: (1) body size, using male carapace width as a proxy; (2) leg length, using the combined length of male tibia 1 + metatarsus 1 as a proxy, because these can be measured easily and precisely and they are available from the taxonomic literature; (3) relative leg length, which is the ratio of leg length to body size; and (4) leg proportion, which is the ratio of male metatarsus 1 to tibia 1; it can be used as a proxy for microhabitat preference (i.e., “space,” “leaf,” and “ground”), in which leaf and space living pholcids tend to have larger leg proportion values (Eberle et al. 2018). The analyses were applied for each of the three selected six-gene trees. Body size and leg length were log-transformed before the analyses (base 2).

#### Speciation and extinction dynamics

We modeled the diversification in pholcids using BAMM (v2.5.0) (Rabosky 2014), because model-based approaches have higher or equivalent accuracy (Jablonski 2017; Title and Rabosky 2019; Cerezer et al. 2023) compared with other methods including the inverse terminal branch lengths (Steel and Mooers 2010), node density metric (Freckleton et al. 2008), and DR statistic (Jetz et al. 2012). We accounted for the influence of incomplete taxon sampling by incorporating genus- and subfamily-specific sampling fractions in our analyses. The total number of species in each group (referred to as the “estimated number”) was estimated using the method described in Eberle et al. (2018) (see Supplementary Table S4). The sampling fraction for each group was then determined by dividing the number of sampled species in the tree by the estimated number. Additionally, for the analysis using genus-specific sampling fractions, we further evaluated the results against the currently known species richness (“Current + Undescribed number”), which includes all described species and undescribed species available in collections and known to BAH (see Supplementary Table S4). In this case, the sampling fraction was calculated by dividing the number of sampled species in the tree by the “Current + Undescribed number.” The prior on diversification shifts was 50, whereas the priors for the lambdaInitPrior, lambdaShiftPrior, and muInitPrior parameters were estimated with BAMMtools (v2.1.10) (Rabosky 2014). For each tree, two independent runs were applied, and for each run, we used 50 million generations for MCMC simulation. Convergence of MCMC chains and effective sampling sizes for all runs were evaluated using the R package coda (v0.19-4) (Plummer et al. 2006). Effective sizes varied from 1000 to 6000 (minimum 440). We used 20% of the generations as burn-in. The single best shift configuration was identified with the function getBestShiftConfiguration() in BAMMtools. Group-specific (i.e., genus and subfamily) speciation rates, extinction rates, and net diversification rates were calculated and we assessed if different groups have different rates using the Kruskal–Wallis H-test in the package pingouin (v0.5.3) (Vallat 2018). We also conducted a rate-through-time analysis using the plotRateThroughTime() function and performed a macroevolutionary cohort analysis with the cohorts() function in BAMMtools.

#### Phylogenetic signal and model fitting of trait evolution

Blomberg’s *K* (Blomberg et al. 2003) and Pagel’s λ (Pagel 1999) of the four traits were assessed using the R package phytools (v1.9-16) (Revell 2012). A series of trait evolution models (BM, OU, EB, rate_trend, lambda, delta, and white) were fitted using the fitContinuous() function in the package geiger. BM (Brownian motion) is a “random walk” model without constraints on rate evolution (Felsenstein 1973); OU (Ornstein–Uhlenbeck) is a BM model with a trend evolving to an optimal trait value (Butler and King 2004); rate_trend is a diffusion model with linear trends in rates through time; EB is BM with an exponential acceleration or deceleration of the evolutionary rate through time (Harmon et al. 2010); and delta is a time-dependent model of trait evolution (Pagel 1999). Lambda measures the extent to which the phylogeny predicts covariance among trait values for species, with λ = 0 corresponding to a star-like phylogeny whereas λ = 1 corresponds to the BM model (Pagel 1999); finally, white is a null model assuming trait data are independent. To find the optimal solution, we applied 30,000 random starting points and all available optimization methods. The optimal models were chosen based on AICc (the sample-size corrected Akaike information criterion).

#### Ancestral state reconstruction

We reconstructed the ancestral states of the four traits using the ace(), anc.ML(), anc.trend(), and anc.Bayes() functions in the packages ape (v5.7-1) (Paradis and Schliep 2019) and phytools based on the available BM, EB, OU, and the mean_trend models.

#### Trait evolution rate estimation

The evolutionary rates of the four traits were estimated with BAMM whose performance is similar to other tools such as BayesTraits (Venditti et al. 2011; Cerezer et al. 2023). The betaInitPrior and betaShiftPrior priors were estimated with BAMMtools, and expectedNumberOfShifts was set to 50. Similar to the speciation/extinction analysis described above, we ran two independent runs for each tree using 50 million generations for MCMC simulation. Convergence and effective sizes were confirmed with the R package coda. To assess the impact of incomplete taxon sampling, we further randomly selected 650 species from the 991 species of the original tree and reran the analysis. Subsequently, subfamily-specific evolutionary rate and evolutionary rate through time plots of the traits were calculated with BAMMtools.

#### Correlation analysis between traits

The phylogenetic generalized least squares (PGLS) analyses were applied to estimate the correlations between different traits using the gls() function in the R package nlme (v3.1-163) (Pinheiro 2023).

#### Trait disparity and diversification

Disparity through time (DTT) for each trait was estimated using the dtt() function in the R package geiger for each subfamily. Trait evolutionary flexibility was also assessed with standard variance of trait values within a group (O’Meara et al. 2006), and we further evaluated the relationships between trait evolutionary flexibility, speciation rate, extinction rate, net diversification rate, trait evolution rate, and estimated species richness using PGLS.

#### Trait-dependent diversification

We tested the correlation between the four traits and pholcid diversification using two methods. (1) The QuaSSE (Quantitative State Speciation and Extinction) model in the R package diversitree (v0.9-20) (FitzJohn 2010), which simultaneously analyzes the relationship between changes in continuous traits and diversification and can suffer from model misspecification and phylogenetic pseudoreplication (Harvey and Rabosky 2018). A global sampling fraction was applied, because the model does not support clade-specific sampling fractions. We fitted the speciation and extinction rates as constant, linear, sigmoidal, and hump-shaped functions of the traits, respectively. We also tested the models without the drift parameter (i.e., assuming a constant mean of trait values through time). Finally, we performed a likelihood ratio test to compare the model fits. (2) The tip-rate correlation *ES-sim* test, which does not require a fully parameterized model of diversification and trait evolution and directly examines the correlation between trait variation across the tips of a phylogeny and tip-specific speciation rate estimates and is thus believed to have rare false inferences (Harvey and Rabosky 2018). We used 20,000 simulations to build the null distribution of trait-speciation associations for significance testing.

## Results

### Pholcidae Phylogeny

A total of 145 Gbp raw data (on average 1.5 Gbp per sample) were generated from the target capture sequencing of the 96 samples (Supplementary Table S1). From these, 10 data matrices were generated for phylogenetic analysis (Supplementary Fig. S1). The “all-blast-all” filtering removed 13.2 Mbp from the 100 samples (including four from NCBI). It is worth noting that blast hits do not necessarily mean real cross-contaminations; they can, for example, represent microbial DNA such as *Wolbachia, Rickettsia*, and *Spiroplasma*, which are known endosymbionts of spiders (Goodacre et al. 2006), or highly conserved genomic regions, such as ribosomal DNA (rDNA) genes. Most samples had little missing data across loci (Supplementary Figs. S2 and S3).

There were 122 loci in matrix 3 (total sites: 32,100 bp; total sequences: 8,704), and the length of the loci ranged approximately from 200 to 500 bp. The saturation analysis shows that 45 loci (36.9%) were saturated (Supplementary Table S5). The assumptions of stationarity and homogeneity for 13 loci (10.7%) were rejected (*P*-values [SymPval] < 0.01) (Supplementary Table S6). The likelihood mapping plot (Supplementary Fig. S4) shows that there are equal numbers of quartets (32.7%) located at the three corners of the triangular graph, indicating the data are suitable to construct phylogeny (Strimmer and von Haeseler 1997). The compositional homogeneity test by IQ-TREE showed that there were only 61 sequences (0.7%) whose character composition significantly deviated from the average composition of the alignment (Supplementary Fig. S5). Similarly, RCFV scores from BaCoCa ranged from 0 to 0.0081 (average: 0.0005), which also indicates a low level of compositional heterogeneity (Kocot et al. 2017) (Supplementary S6). However, taxon-wise, the RCFV scores (0.0168–0.0695) of all taxa were quite high, indicating a high level of compositional heterogeneity (Supplementary Table S7). The intra-locus recombination analysis (*P*-value < 0.05) identified 483 recombinant sequences (5.5% of total sequences) involving 52 loci (42.6%); however, there were only 36 recombinants involving 7 loci (5.7%) with a *P*-value < 0.01 and longer than 100 bp (Supplementary Table S8).

**Figure 1.**
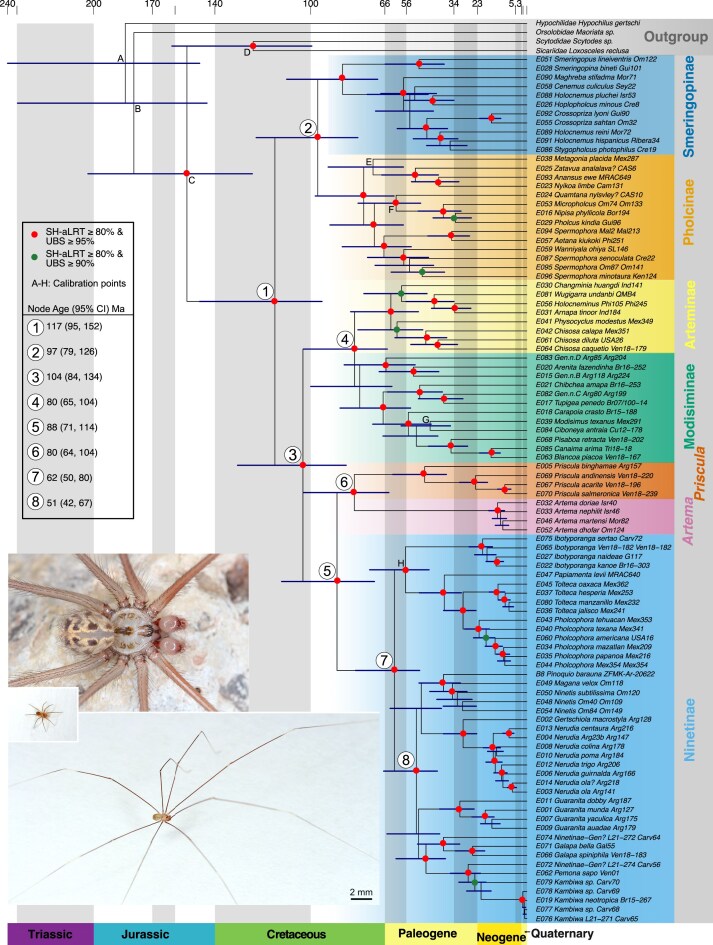
Maximum likelihood phylogenetic tree and divergence times of Pholcidae. The tree was constructed with IQ-TREE and data matrix 3 using a partitioned model in which all loci share the same set of branch lengths, whereas each locus has its own evolution rate. Branch supports were evaluated with 2000 ultrafast bootstrap (UBS) and SH-aLRT branch test based on 2000 bootstrap replicates. MCMCTree program in PAML and node-dating strategy were applied to calibrate the divergence times based on fossil records. Images of spiders (taken by BAH) were brought to the same scale, illustrating ranges in body size and relative leg length in Pholcidae; from top to bottom: *Artema martensi; Galapa gabito* (Ninetinae); *Litoporus* sp. “Br16-129” (Modisminae).

All analyses except those based on matrices 9 and 10 recovered the same topology in terms of subfamily-level phylogenetic relationships, and very similar intergeneric and interspecific relationships. An overview of tree topology consistency across all analyses and matrices can be found in Supplementary Figure S6, whereas Supplementary Figures S7–S10 show the trees (based on matrix 3) from RAxML-NG, PhyloBayes-MPI, ASTRAL, and SVDquartets. The unconstrained tree inferences based on UCEs and six-gene matrices also display similar topologies. Notably, all misplaced taxa were represented by CO1 only (Supplementary Data 2). We chose the tree from IQ-TREE partitioned analysis based on matrix 3 as our preferred tree (Fig. 1), whose branches all show high gene frequency supports (≥43%) (Supplementary Fig. S11a,b). The analyses based on matrices 9 and 10 (i.e., MSAs consisting of compositionally homogenous sites only) failed to recover monophyletic subfamilies due to too few sites left (not shown).

Our phylogeny (Fig. 1) concurs with previous studies (Bruvo-Mađarić et al. 2005; Huber 2011; Dimitrov et al. 2013; Eberle et al. 2018; Huber et al. 2018) that were based on a few genes or a combination with morphological characters in terms of the following subfamily-level relationships: (1) Smeringopinae is sister to Pholcinae (gene frequency support: 63%) and (2) Modisiminae is sister to Arteminae (gene frequency support: 79%).

However, our study suggests that (1) the traditional Arteminae is not monophyletic but contains two independent lineages, *Artema* and “other Arteminae,” which is also supported by the topology test analysis (Supplementary Table S9). (2) *Priscula* is not part of Modisiminae but sister to *Artema* (gene frequency support: 43%). (3) The subfamily Ninetinae that contains some of the tiniest Pholcidae is not sister to all other pholcids but to *Artema* + *Priscula*, two genera that contain some of the largest Pholcidae (gene frequency support: 65%), as evidenced by the topology test analysis (Supplementary Table S9). There are 61.3% of quartets supporting the topology ([Ninetinae, [*Artema, Priscula*]], [Arteminae, Modisiminae]) in the likelihood mapping analysis (Supplementary Fig. S11c). Ninetinae has been an enigmatic subfamily. Our large sample (48 species) provides not only a new hypothesis about their position among Pholcidae but also a first hypothesis on intergeneric relationships (Fig. 1).

The most recent common ancestor (MRCA) of pholcids dates back to 117 Ma (95% confidence interval: 95, 152) (Fig. 1), and then it diverged into two major groups. The first group consists of Pholcinae and Smeringopinae, which diverged from each other 97 Ma (79, 126). The second group includes all the remaining pholcids. Arteminae is sister to Modisiminae, and they diverged 80 Ma (65, 104). The (Ninetinae, [*Artema, Priscula*]) group split from (Arteminae, Modisiminae) 104 Ma (84, 134). The (*Artema, Priscula*) group split from Ninetinae 88 Ma (71, 114), whereas *Artema* and *Priscula* diverged from each other 80 Ma (64, 104).

The phylogenetic trees based on *12S, 16S, 18S, 28S, H3*, and *CO1* genes using constrained searches are shown in Supplementary Figures S12–S14. The differences between them include: (1) the number of specimens placed in wrong subfamilies; monophyly of uncontested genera recovered or not; (2) morphologically supported sister-group relationships of genera either recovered or not; and (3) individual species misplaced in wrong genera. These differences come from different alignment trimming strategies. We focus on the question about whether the main results from our comparative analyses are stable using these different topologies, rather than on the possible causes for variation among topologies and on judging which topology is the “best.”

### Speciation and Extinction Rates

**Figure 2.**
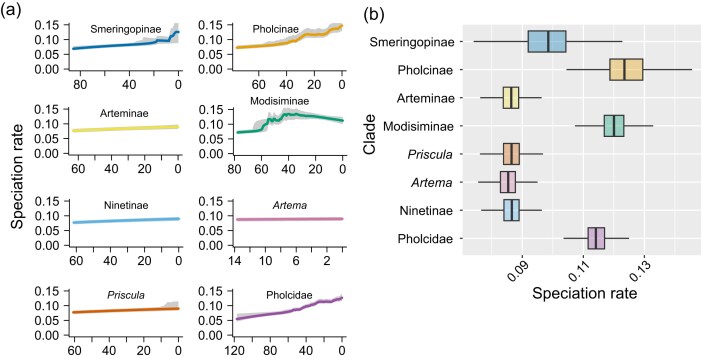
Speciation rate dynamics. Group-specific speciation rates were extracted from the BAMM analysis, based on genus-specific sampling fraction, estimated species richness (see main text and Supplementary Table S4 for details), and the ClipKIT smart-gap tree. (a) Speciation rate through time. Smeringopinae, Pholcinae, and Modisiminae show a rapid increase of speciation rates after their emergence, compared with Arteminae, Ninetinae, *Artema*, and *Priscula*. (b) Boxplot of speciation rates. Pholcinae and Modisiminae show the highest average speciation rates.

BAMM diversification analyses based on the three selected six-gene trees revealed similar patterns, with only four rate shifts (Supplementary Fig. S15), suggesting that speciation rate shifts are unlikely to be the primary factor contributing to the uneven levels of species richness among subfamilies. In the analyses based on estimated species richness (Supplementary Fig. S15a), two speciation rate shifts within the subfamily Pholcinae were identified. The first rate shift involves a clade containing 60% of described Pholcinae, and the second shift occurs within that clade. A third rate shift occurs within the genus *Smeringopina* of the subfamily Smeringopinae, and a fourth rate shift occurs within the clade that contains 91% of described Modisiminae species. The BAMM diversification analyses based on currently known species richness showed similar patterns, except that there were two rate shifts for the genus *Pholcus*, and no rate shift in the subfamily Smeringopinae (Supplementary Fig. S15b). At the subfamily level, Pholcinae, Modisiminae, and Smeringopinae consistently showed higher speciation rates (Pholcinae > Modisiminae > Smeringopinae) than Arteminae, Ninetinae, *Artema*, and *Priscula* (*P*-value << 0.001; Fig. 2b; Supplementary Fig. S16 and Supplementary Table S10). Speciation rate generally increased through time (Fig. 2a). Pholcinae, Modisiminae, and Smeringopinae also showed higher extinction rates (Pholcinae > Smeringopinae > Modisiminae) than the other groups (*P*-value << 0.001; Supplementary Fig. S17 and Supplementary Table S10). The same pattern also applies to the net diversification rates (i.e., speciation—extinction) (Modisiminae > Pholcinae > Smeringopinae) (*P*-value << 0.001; Supplementary Fig. S18 and Supplementary Table S10).

### Trait Evolution

Both Blomberg’s *K* (0.64 ≤ *K* ≤ 1.5, *P*-values = 0.001) and Pagel’s λ (0.95 ≤ λ ≤ 0.99, *P*-values << 0.001) showed high phylogenetic signal for the four analyzed traits, indicating that closely related taxa tend to exhibit similar traits as a result of shared ancestry (Supplementary Fig. S19). Both measures show that leg length has the highest phylogenetic signal, indicating that leg length is a conservative trait. Body size and relative leg length were much more variable.

Our model-fitting analyses for the four traits consistently support the idea that these morphological traits evolve following a Brownian motion-like dynamics. The best-fitted model was rate_trend, followed by EB, then BM or delta, with OU, lambda, and finally white as the least fitted (Supplementary Table S11). Both rate_trend and EB models are extended BM models, which are characterized by an increasing or decreasing rate through time (linear or exponential). For all traits, both the parameter *slope* of the rate_trend model and the parameter α of the EB model were negative, and the parameter δ of the delta model was < 1, indicating the trait evolution rates decrease through time. On the other hand, for all fitted models, the parameters σ^2^ (i.e., the diffusion rate of trait changes) were very small (e.g., σ^2^ ≤ 0.01 for the BM model) except for the trait relative leg length (σ^2^ = 0.51 for the BM model), indicating that relative leg length shows substantial change over time, leading to greater variability among species, whereas the other three traits exhibited relatively limited variability throughout the course of evolution.

**Figure 3.**
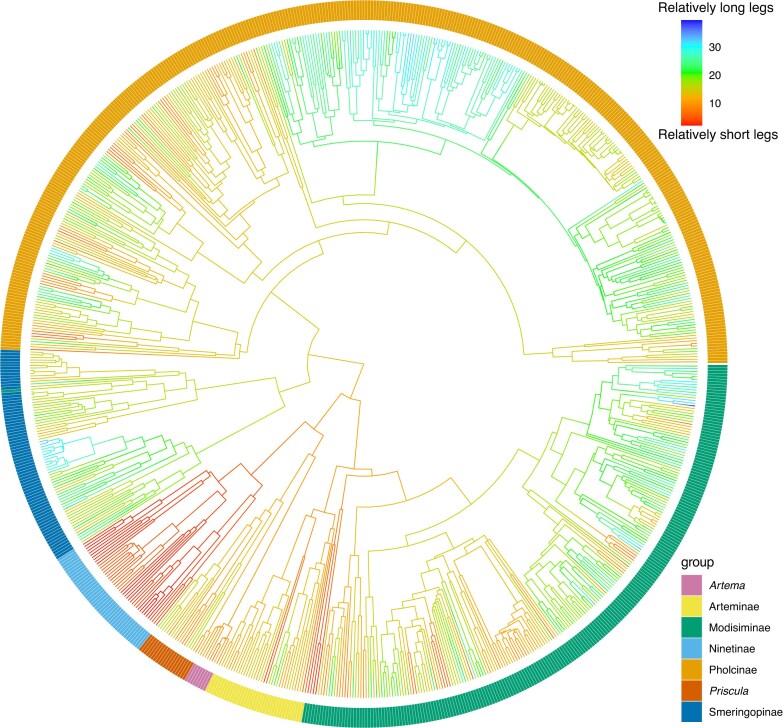
Ancestral state reconstruction. The data on relative leg length are presented; the ClipKIT smart-gap tree was used for analysis. The anc.ML() function in phytools was used to estimate ancestral states (the inner tree), with cooler (blue) color indicating relatively long legs and warmer (red) color indicating relatively short legs. Smeringopinae, Pholcinae, and Modisiminae show higher level of heterogeneity than Arteminae, Ninetinae, *Artema*, and *Priscula* in terms of relative leg length.

**Figure 4.**
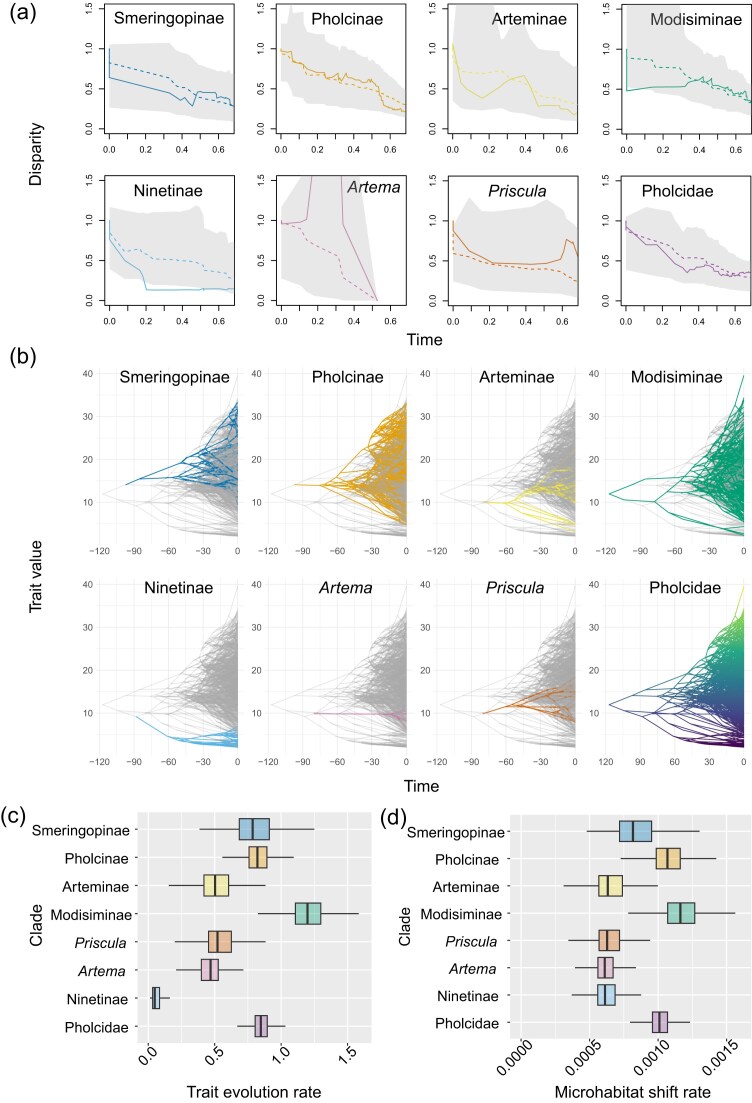
Trait evolution dynamics. The data on relative leg length (a, b, and c) and leg proportion (d) are shown, and the ClipKIT smart-gap tree was used for analysis. (a) Disparity dynamics, estimated by the dtt() function in geiger. The time (x-axis) is relative to a total tree length of 1, which ranges from 0 (root) to 1 (tips). Smeringopinae, Pholcinae, and Modisiminae show higher disparity, compared with Arteminae, Ninetinae, and *Priscula*. The high disparity in *Artema* could be an artifact due to its small sample size (*n* = 9). (b) Phenograms for different groups, based on the BM model and the anc.ML() function in phytools. Smeringopinae, Pholcinae, and Modisiminae show much wider ranges of trait values than Arteminae, Ninetinae, *Artema*, and *Priscula*. (c and d) Group-specific trait evolution rates, extracted from the BAMM trait analysis, which was based on genus-specific sampling fraction and estimated species richness; see main text and Supplementary Table S4 for details. Smeringopinae, Pholcinae, and Modisiminae show higher evolution rates for relative leg length and leg proportion than Arteminae, Ninetinae, *Artema*, and *Priscula*. Leg proportion in Panel d is a proxy of microhabitat preference (Eberle et al. 2018); the evolution rate of leg proportion can thus be interpreted as a microhabitat shift rate.

Overall, the reconstructed ancestral states of the four traits from different models and methods showed heterogeneous patterns of trait values for all groups (except for Ninetinae, *Artema*, and *Priscula*). The pholcid ancestors had intermediate values for the traits, and then the trait values fluctuated in different lineages (Fig. 3; Supplementary Figs. S20–S22). Evolution in Ninetinae is skewed toward small values, whereas *Artema* and *Priscula* evolved toward large values for both body size and leg length; other groups generally evolved toward a much wider range of current values (Fig. 4b; Supplementary Figs. S23–S25).

The subfamily Ninetinae is noteworthy for having the smallest and most homogeneous values for all four traits among pholcids, especially for relative leg length (Figs. 3 and 4a,b; Supplementary Fig. S26). Pholcinae, Modisiminae, and Smeringopinae occupy a broad morphospace and show large disparity (standard variance: 5.79, 5.82, and 5.42, respectively, for relative leg length), whereas the morphospace of Arteminae, Ninetinae, *Artema*, and *Priscula* is more limited (standard variance: 3.17, 1.15, 1.24, and 2.71, respectively) (Figs. 3 and 4a; Supplementary Fig. S26).

Evolutionary rates of the four traits calculated using BAMM based on different trees and different taxonomic sampling were largely consistent (Supplementary Fig. S27), indicating that estimates of rates were not significantly impacted by the uncertainty in tree topology or sampling fractions. Specifically, for both relative leg length and leg proportion, Smeringopinae, Pholcinae, and Modisiminae consistently showed the highest rates, whereas Ninetinae showed the lowest rate for relative leg length, and Arteminae, Ninetinae, *Artema*, and *Priscula* showed similar low rates for leg proportion (Fig. 4c,d).

Similar to Eberle et al. (2018), we found that space- and leaf-dwelling spiders tended to have larger leg proportion values (Supplementary Fig. S28a). Space- and ground-dwelling spiders tended to have larger body sizes than leaf dwellers, whereas space and leaf dwellers tended to have longer legs than ground spiders (Supplementary Fig. S28b,c). On the other side, all pholcids, regardless of habitat, had a wide range for relative leg length (Supplementary Fig. S28d).

**Figure 5.**
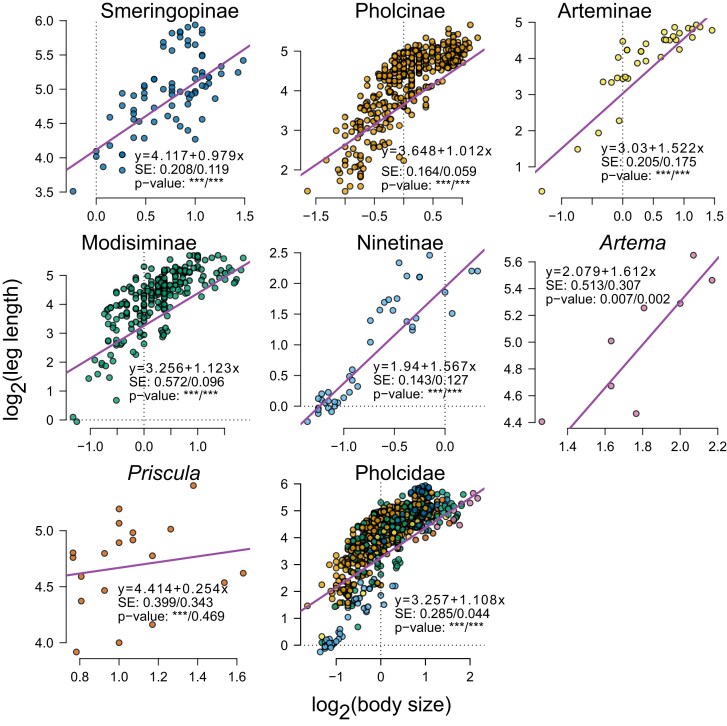
Evolutionary allometry of pholcids. Data points for each group are shown in different colors. Relationships between log_2_(*leg length*) and log_2_(*body size*) were determined by phylogenetic generalized least squares regression. SE: Standard error. ***P-value << 0.001. The two items separated by “/” are SE and *P*-values for the intercept and slope, respectively. Leg length generally showed a positive allometry to body size in all groups but with varied slopes and intercepts.

PGLS analysis based on the three six-gene trees consistently found a linear correlation between body size and leg length for most groups (Fig. 5). Overall, Pholcidae species showed a slightly positive allometry in terms of leg length (slope ± standard error [SE]: 1.108 ± 0.044, *P*-value << 0.001). Interestingly, the species-diverse groups Pholcinae, Modisiminae, and Smeringopinae all showed a trend similar to isometry with slopes of 1.012 ± 0.059, 1.123 ± 0.096, and 0.979 ± 0.119 (*P*-values << 0.001), respectively, whereas the species-poor groups Arteminae, Ninetinae, and *Artema* showed a strong positive allometry with slopes of 1.522 ± 0.175, 1.567 ± 0.127, and 1.612 ± 0.307 (*P*-values for Arteminae and Ninetinae << 0.001, for *Artema:* 0.002), respectively. *Priscula* showed a non-significant negative allometry (slope: 0.254 ± 0.343, *P*-value: 0.469). Regarding intercepts, Pholcinae, Modisiminae, Smeringopinae, Arteminae, and *Priscula* had similar, large values (3.648 ± 0.164, 3.256 ± 0.572, 4.117 ± 0.208, 3.03 ± 0.205, and 4.414 ± 0.399, *P*-values: << 0.001), whereas Ninetinae and *Artema* had similar but smaller values (1.94 ± 0.143, 2.079 ± 0.513, *P*-values: << 0.001 and 0.007, respectively) (Fig. 5). Body size, leg length, or relative leg length all showed a linear correlation with leg proportion (Supplementary Fig. S29).

### Relationships between Trait Variation and Diversification

The tip-rate correlation test (Supplementary Table S12) showed that body size and leg length are positively related to net diversification rates (Pearson’s correlation coefficient ρ = 0.34 and *P*-value < 0.05), whereas the correlation with relative leg length or leg proportion was not significant (Pearson’s correlation coefficient ρ = 0.24 and *P*-value > 0.05). The QuaSSE analysis, on the other hand, showed that speciation and extinction rates are a sigmoidal or hump function of the traits (Supplementary Table S13).

**Figure 6.**
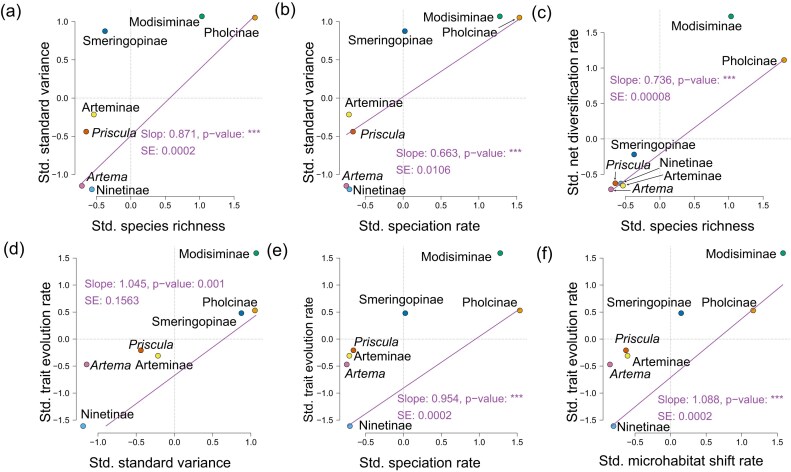
Relationships between disparity, species richness, trait evolution, diversification, and microhabitat shift. Relative leg length and the ClipKIT smart-gap tree were used for analysis. Disparity was quantified as the standardized standard variance of the trait (the *y*-axis in Panels a and b), which was calculated by combining the trait values of extant taxa (tips) and the ancestral trait states reconstructed using the Brownian motion model. The estimated species richness for each group was used in the analysis (a and c); see the main text and Supplementary Table S4 for details. Trait evolution rates (the *y*-axis in Panels d, e, and f) were estimated by BAMM. Leg proportion is a proxy of microhabitat preference (Eberle et al. 2018); the evolution rate of leg proportion (estimated by BAMM) can thus be seen as a microhabitat shift rate (the *x*-axis in Panel f). The relationships between these variables were determined by phylogenetic generalized least squares regression. Std.: Standardized. SE: Standard error of the slope. ***P -value << 0.001.

The PGLS analysis showed that the amount of disparity (standard variance) of traits is significantly positively correlated with species richness and speciation rate (Fig. 6a,b; Supplementary Fig. S30). For example, relative leg length had a correlation of slope with species richness = 0.871 (*P*-value << 0.001) (Fig. 6a), whereas it had a correlation of slope with speciation rate = 0.663 (*P*-value << 0.001) (Fig. 6b). Pholcinae, Modisiminae, and Smeringopinae had the greatest variance and highest speciation rates, whereas Arteminae, Ninetinae, *Artema*, and *Priscula* had the opposite. Pholcinae and Modisiminae are the pholcids with the largest net diversification rates (speciation—extinction) and highest species richness (Fig. 6c). Finally, the trait evolution rate of relative leg length is positively correlated with disparity, speciation rate, and the rate of microhabitat shift (slopes ± SE: 1.045 ± 0.1563, 0.954 ± 0.0002, 1.088 ± 0.0002, *P*-values: << 0.001, << 0.001, and 0.001, respectively) (Fig. 6d–f).

## Discussion

### A Robust Phylogeny for Pholcidae

Based on a sample of 96 taxa covering all subfamilies of Pholcidae, and using UCE target capture, we obtained the largest number of loci (122) to date for pholcids. Because our phylogeny conflicts with previous molecular and morphological data (see below), we did an extensive search for possible errors. The outcome of phylogenetic analysis can be affected by various factors, such as contamination, model misspecification, missing data, incomplete lineage sorting (ILS), and introgression (Zhang et al. 2018; Tiley et al. 2023). We scrutinized cross-contamination by filtering out potential cross-contaminant sequences using the “all-blast-all” method with sequence abundance, similarity (≥ 99%), and alignment length (≥ 100 bp) being considered. We also tried alternative blast similarity cutoffs (≥ 98% or ≥ 97%) (not shown), but the results of phylogenetic analyses (i.e., the subfamily-level relationships) remained the same, indicating a good quality of our data set. Our data set appears unaffected by compositional heterogeneity. Only 61 sequences (0.7%) showed character composition deviating from the average composition of the alignment, as indicated by the compositional homogeneity test (Supplementary Fig. S5). Additionally, the RCFV measurement values were very low (0–0.0081, average 0.0005) (Kocot et al. 2017) (Supplementary Table S7). Indeed, both the RY-coding strategy-based phylogeny (matrix 8; Supplementary Fig. S6), which aims to reduce to the impact of compositional heterogeneity on phylogenetic inference (Criscuolo and Gribaldo 2010), and the compositional heterogeneity-aware model CAT-GTR+G4 in PhyloBayes-MPI (matrix 3; Supplementary Fig. S7) yielded topologies similar to those in Figure 1. The intra-locus recombination analysis identified only 36 recombinants involving seven loci (5.7%) with a *P*-value < 0.01 and longer than 100 bp (Supplementary Table S8), suggesting intra-locus recombination should not be a severe problem either, and our coalescent analyses (i.e., ASTRAL) gave a similar result (Supplementary Fig. S9). Most quartets (98.1%) were distributed at the three corners of the triangular graph in the likelihood mapping analysis, indicating a high level of phylogenetic signal (Supplementary Fig. S4). We also assessed the influences of different MSA trimming programs (Gblocks vs. TrimAl) (Supplementary Fig. S1a); however, all data matrices resulted in the same subfamily-level relationships. Overall, our UCE data sets are of high quality and appropriate for phylogenetic analysis. All partitioned ML analyses (IQ-TREE vs. RAxML-NG; Fig. 1 and Supplementary Fig. S7), Bayesian multispecies coalescence (MSC) analysis (PhyloBayes-MPI; Supplementary Fig. S8), and summary-based MSC analyses (ASTRAL [Supplementary Fig. S9] and SVDquartets [Supplementary Fig. S10]) consistently recovered the same subfamily-level topology, and very similar genus- and species-level relationships (Supplementary Fig. S6), indicating our phylogeny is robust.

Our phylogeny supports numerous previously hypothesized relationships, resolves dubious nodes, and solves several open questions. At subfamily level, the sister-group relationships Pholcinae + Smeringopinae and Modisiminae + Arteminae have been proposed before (Dimitrov et al. 2013; Eberle et al. 2018) and receive additional strong support (gene frequency support: 63% and 79%, respectively; Supplementary Fig. S11a,b). The separation of *Artema* from “other Arteminae” was first proposed by Eberle et al. (2018) and is also strongly supported by our new data (gene frequency support: 79%; Supplementary Fig. S11a,b). The most fundamental changes to previous ideas concern three taxa that were identified as the “most obvious gaps” in understanding pholcid phylogeny (Huber et al. 2018): Ninetinae, *Artema*, and *Priscula*. Our data strongly suggest that these three taxa together constitute a monophyletic group: (Ninetinae, [*Artema, Priscula*]) (gene frequency support: 65%; Supplementary Fig. S11a,b), whereas the sister-group relationship of *Artema* and *Priscula* is supported by 43% of the genes and by 61% of the sites (Supplementary Fig. S11c).

This topology is difficult to reconcile with morphological evidence, particularly in relation to sperm ultrastructure and genital mechanics. Pholcidae are part of a large clade of spiders characterized by a specific sperm transfer form, where sperm are united in packages (synspermia) (Michalik and Ramírez 2014). However, most Pholcidae have switched to another sperm transfer form: they transfer sperm individually (cleistospermia) (Dederichs et al. 2022). Synspermia in Pholcidae have only been found in certain Ninetinae, which has been interpreted as a retention of the ancestral state, supporting a “basal” position of Ninetinae, as sister to all other Pholcidae (Dederichs et al. 2022). Our phylogeny suggests that synspermia have either been regained in Ninetinae or transformed to cleistospermia four times independently within Pholcidae. Details of genital mechanics, on the other hand, have been considered as strong evidence for the monophyly of traditional Arteminae (i.e., *Artema* + “other Arteminae”). All traditional Arteminae share a pair of unique structures on each male intromittent organ (procursus) that allows for an asymmetric insertion of right and left procursi during copulation (Huber and Eberhard 1997; Aharon et al. 2017). Our phylogeny suggests that this complex and seemingly identical mechanism has either evolved independently in *Artema* and “other Arteminae” or that it has been lost at least three times independently. Interestingly, the monophyly of *Artema* + “other Arteminae” has previously been supported by molecular data (*12S*/*16S*/*18S*/*28S*/*H3*/*CO1*) (Dimitrov et al. 2013). Finally, we note that we are not aware of any specific morphological similarity between *Priscula, Artema*, and Ninetinae that is likely to constitute a synapomorphy.

Our estimates of divergence times (Fig. 1) are consistently younger than those of a previous study (Dimitrov et al. 2013). For instance, we recovered the MRCA of Pholcidae to be 117 (95, 152) Ma, whereas Dimitrov et al. (2013) recovered 207 (185, 228) Ma. A more recent study by Magalhaes et al. (2020) highlighted that over half (54%) of fossil calibrations in previous spider studies were problematic. By critically curating key fossil records and integrating transcriptomic data, they produced a revised dated spider tree. Their findings suggest that Pholcidae originated no earlier than 145 Ma, with a range of 80–200 Ma, which is congruent with our estimate of 117 Ma for the divergence of Pholcidae.

### Subfamily Species Diversity and Morphological Trait Evolution in Pholcids

Pholcids exhibit variable morphological traits, with body sizes ranging from 1 to 10 mm, and leg spans from 3 to 180 mm. Within Pholcidae, Pholcinae and Modisiminae (excl. *Priscula*) are the two largest subfamilies, with currently (May 2025) 1150 and 510 described species, respectively (B.A. Huber, unpubl. data). Their diversity is largely concentrated in humid tropical and subtropical regions. Smeringopinae, the third-largest subfamily, includes 159 described species. It is often found in semi-arid regions, except for the tropical African genus *Smeringopina* Kraus, 1957 (Huber et al. 2018). The less diverse groups are Arteminae (107), Ninetinae (80), and *Artema* + *Priscula* (40). Among the species-poor groups, Ninetinae, Arteminae, and *Artema* are mostly or strictly distributed in rather dry regions (Huber et al. 2018; Huber 2021), whereas *Priscula* is largely restricted to high-elevation Andean forests (Huber, Meng, Dupérré, et al. 2023).

What are the drivers behind the high species diversity, speciation, and net diversification rates in Pholcinae and Modisiminae, as shown in Figures 2b and 6c? One indirect but intuitive explanation refers to the complexity of the environments these spiders inhabit, combined with their ability to adapt to various microhabitats (Eberle et al. 2018). Eberle et al. (2018) demonstrated frequent microhabitat shifts throughout pholcid evolution, with many back-and-forth transitions between near-ground habitats and leaves, particularly in the two largest subfamilies, Pholcinae and Modisiminae. Additionally, leaf- and space-dwelling species were found to have higher diversification rates. Thus, high evolutionary flexibility in microhabitat use may have played a key role in driving pholcid diversification. Our analysis further supports this idea. Leg proportion can be seen as a proxy for different types of microhabitats (i.e., “space,” “leaf,” and “ground”), and space and leaf dwellers tend to have larger values of leg proportion (Eberle et al. 2018). We found that Modisiminae, Pholcinae, and Smeringopinae have higher trait evolution rates for leg proportion, indicating these groups have experienced more frequent microhabitat transitions (Fig. 4d), and indeed, the extent of flexibility in microhabitat use is positively correlated with species richness (Supplementary Fig. S30c, left).

Understanding the factors that shape phenotypic differences among lineages is also crucial for explaining the variation in species richness across different lineages. Overall, the four analyzed traits showed a high level of phylogenetic signal (0.64 ≤ Blomberg’s *K* ≤ 1.5, *P*-values = 0.001; 0.95 ≤ Pagel’s λ ≤ 0.99, *P*-values << 0.001), indicating closely related species are prone to resemble each other for these traits. Leg length was the most conserved of the four traits, as indicated by its high values for both Blomberg’s *K* (1.5, *P*-value = 0.001) and Pagel’s λ (0.99, *P*-value << 0.001), reflecting minimal deviation from evolutionary expectations. In contrast, relative leg length was the most variable trait, with lower values for Blomberg’s *K* (0.76, *P*-value = 0.001) and Pagel’s λ (0.95, *P*-value << 0.001), suggesting greater evolutionary lability. This coincided with our model-fitting analysis. For all fitted models, the diffusion rates, σ^2^, were found to be remarkably small. Most traits had a σ^2^ ≤ 0.01; only the trait relative leg length had a higher σ^2^ = 0.51. This indicates that most traits evolve much slower than relative leg length.

Our analysis also shows that the four analyzed traits are consistently best-fitted by Brownian motion-like models including rate_trend and EB, then BM or delta (Supplementary Table S11), and estimates of related parameters (both slope and α < 0, δ < 1) of these models suggest that trait evolution rates are decreasing through time. A possible explanation is that there were few competitors when pholcids entered new adaptive zones, leading to diversification and increased phenotypic diversity. However, as the available ecological space filled up and competition increased, the rate of morphological evolution naturally slowed down. The need for further rapid changes diminished because the organisms had already adapted to their environments, leading to a deceleration in the pace of trait evolution over time (cf. Simpson 1984; Losos and Miles 2002; Harmon et al. 2010).

#### Body Size

Wolff et al. (2022) suggested that most morphological traits in spiders, including body size, do not significantly vary across ecological guilds. Our analysis adds a temporal perspective (Supplementary Fig. S23). Although the body size of all pholcids evolved in a Brownian-like motion, distinct subgroups exhibited unique patterns: Smeringopinae, *Artema*, and *Priscula* showed a trend toward larger body sizes, supporting Cope’s rule, whereas Ninetinae exhibited a shift toward smaller sizes, contradicting Cope’s rule. Meanwhile, Modisiminae, Pholcinae, and Arteminae evolved in line with the Brownian motion model. It has been suggested that web-building spiders tend to have smaller body sizes, whereas hunting spiders are generally larger (Wolff et al. 2022; Shao et al. 2023). Our study provides more detail on body size variation within web-building spiders. Benefits of evolving toward large body size for *Priscula, Artema*, and Smeringopinae may include: (1) enhanced protection against predators and improved prey capture due to increased strength; (2) increased mating success through sexual selection; and (3) higher fecundity (Wells 1988; Kuntner and Coddington 2020). On the other side, adult and juvenile viability selection (Blanckenhorn 2000) could be responsible for Ninetinae evolving toward small body size. Thus, gigantism and dwarfism may represent two distinct strategies employed within Pholcidae to reduce competition with close relatives with intermediate body size (Supplementary Figs. S20 and S23).

#### (Relative) leg length

The evolution of leg length in pholcids mirrors the patterns observed in body size; however, relative leg length showed different trends. Although the evolution of relative leg length in most groups follows a Brownian motion-like pattern, Smeringopinae exhibit a slight shift toward longer relative leg lengths. The most intriguing group, however, is Ninetinae, which evolves toward an extremely small relative leg length. This aligns with their ground-dwelling habits and fast-running behavior (Huber, Meng, Král, et al. 2023; Huber, Meng, Valdez-Mondragón, et al. 2023; Huber, Meng, García, et al. 2024; Huber, Meng, Dederichs, et al. 2024). Similar patterns have also been observed among spiders in general. For instance, aerial web builders generally have longer legs compared with substrate-bound web builders and ground runners (Wolff et al. 2022).

#### Phenotypic flexibility

Although the direction of trait evolution (e.g., Cope's rule) may play a role in species diversification, the significance of variation in trait values across time (referred to here as evolutionary flexibility) has received less attention. The reconstructed ancestral states of body size and (relative) leg length (Fig. 3; Supplementary Figs. S20 and S21) clearly demonstrate the high degree of flexibility in these traits, as evidenced by the significant heterogeneity in trait values, especially in relative leg length (Fig. 3). Our analyses also clearly indicate that different subfamilies exhibit varying degrees of phenotypic flexibility. The species-rich subfamilies (Modisiminae, Pholcinae, and Smeringopinae) consistently showed higher levels of phenotypic flexibility compared with the species-poorer subfamilies (Arteminae, Ninetinae, *Artema*, and *Priscula*) (Fig. 3; Supplementary Figs. S20 and S21). Because body size and relative leg length are key traits influencing species' interactions with their biotic and abiotic environments, the observed differences in flexibility and species richness among subfamilies suggest that phenotypic flexibility may play a critical role in pholcid diversification. This flexibility could enable species to explore a broader morphospace, facilitating adaptation to diverse environmental conditions and potentially driving evolutionary diversification. Indeed, a recent study on the mantidflies (Neuroptera: Mantispoidea) also highlighted the importance of evolutionary flexibility of traits in maintaining or accelerating species diversification (Li et al. 2024). We will detail this in the next section.

### Evolutionary Flexibility Is Positively Correlated with Trait Evolution Rate and Speciation Rate

To quantify phenotypic flexibility, we measured disparity and standard variance of traits (O’Meara et al. 2006). Our results show that the species-rich subfamilies Pholcinae, Modisiminae, and Smeringopinae exhibit larger standard variances for relative leg length (5.79, 5.82, and 5.42, respectively), compared with the species-poorer subfamilies Arteminae, Ninetinae, *Artema*, and *Priscula*, which have much lower standard variances (3.17, 1.15, 1.24, and 2.71, respectively) (Fig. 4a,b). Similar results were obtained in the analyses based on the three six-gene trees, on different trait evolutionary models (BM, OU, trend) and on different traits (body size, leg length) (Supplementary Figs. S23 and S24), suggesting that our estimate of flexibility is robust.

Our PGLS analysis shows that the standard variance of the studied traits is positively correlated with species richness and speciation rates (slopes: 0.871 ± 0.0002, 0.663 ± 0.0106, *P*-value: << 0.001, respectively) (Fig. 6a,b). The higher species diversities and speciation rates in Modisiminae, Pholcinae, and Smeringopinae are accompanied with higher phenotypic flexibility and larger net diversification rates (Fig. 6a–c). Further analysis showed that phenotypic flexibility was positively correlated with trait evolution rate (Fig. 6d), whereas the latter was also positively correlated with speciation rate (Fig. 6e). For instance, Modisiminae, Pholcinae, and Smeringopinae showed higher evolution rates for relative leg length than Arteminae, Ninetinae, *Artema*, and *Priscula* (Fig. 6e). It could be argued that differences in extinction rates have led to variations in species diversity among Pholcidae subfamilies. However, our analysis shows that Modisiminae, Pholcinae, and Smeringopinae all exhibit higher extinction rates than the remaining groups (Supplementary Fig. S17). This suggests that differences in speciation rates rather than extinction rates are the primary factor shaping species diversity patterns among pholcid subfamilies. However, it should be noted that the speciation rates in our study were estimated using BAMM with clade-specific sampling fractions, a practice that has been criticized for potentially yielding incorrect likelihoods in some scenarios (Laudanno et al. 2021). In our analyses, BAMM was used as a descriptive tool to summarize relative differences among clades, and we refrained from interpreting absolute speciation or extinction rate estimates, or from making inferences about the precise placement of rate shifts.

Two key aspects of our results should be emphasized, regarding sister groups with the same evolutionary age: (1) A greater net diversification rate—whether driven by a higher speciation rate and/or a lower extinction rate—leads to increased species diversity (Fig. 6c), and (2) a higher rate of trait evolution results in greater phenotypic diversity (Fig. 6d). Pholcinae + Smeringopinae and Modisiminae + Arteminae form two sister groups (Fig. 1). Both Pholcinae and Modisiminae exhibit greater species richness, higher speciation rates, and increased phenotypic diversity than their sister taxa Smeringopinae and Arteminae, respectively. This pattern of convergent evolution suggests that increased phenotypic diversity and accelerated trait evolution rate have likely played a significant role in the diversification of pholcids. Indeed, a positive correlation between trait evolution rate and speciation rate has also been observed in several recent studies of vertebrates, including hummingbirds (plumage color evolution) (Beltran et al. 2021) and fishes (body size evolution) (Rabosky et al. 2013; Cerezer et al. 2023). This positive correlation generally holds true at broad macroevolutionary scales for body size evolution, which has been tested in the five major vertebrate clades (Cooney and Thomas 2021): amphibians, birds, mammals, ray-finned fish, and squamate reptiles; but negative correlation has also been found in clades including *Bufo* toads (amphibians) and lacertid lizards (squamates). Furthermore, non-significant relationships are also observed in many subgroups (Cooney and Thomas 2021). It should be noted, however, that phenotypic diversification can occur after, alongside, or even before species diversification (Jablonski 2017; Folk et al. 2019). As such, additional evidence from biogeography, ecology, genetics, and other aspects is required to establish causality.

The higher trait evolution rate in Modisiminae, Pholcinae, and Smeringopinae indicates they exhibit a higher evolutionary potential to produce diverse phenotypes, which in turn are adaptations to corresponding environments. Evolutionary constraints may limit the evolutionary potential (Arnold 1992). In the phenograms of the four traits (Fig. 4b; Supplementary Figs. S23–S25), these three subfamilies occupy much wider morphospace, whereas the other four species-poor subfamilies (Arteminae, Ninetinae, *Artema*, and *Priscula*) occupy much narrower morphospace. Additional support for this finding comes from the fact that the majority of troglomorphic pholcids are found in two subfamilies, Modisiminae and Pholcinae, with only a few slightly troglomorphic species in Smeringopinae, *Artema*, Arteminae, and *Priscula*, and none in Ninetinae (Huber 2018). Furthermore, our data support the habitat heterogeneity hypothesis, which claims that increased environmental heterogeneity promotes greater species diversity by offering more ecological niches (Allouche et al. 2012). Indeed, we found that Modisiminae, Pholcinae, and Smeringopinae have greater microhabitat shift rates—measured using the evolution rate of leg proportion as a proxy (Fig. 6f)—consistent with numerous microhabitat shifts found in these subfamilies by Eberle et al. (2018). Our estimation of microhabitat shift rates is conservative, as the substantial overlap in leg proportion between leaf-dwelling and space-living species (Supplementary Fig. S28a) may have led to an underestimation of the actual frequency of microhabitat shifts. Modisiminae and Pholcinae are mostly found in humid tropical and subtropical regions, and their varied traits are thought to be adapted to the wide range of forest microhabitats (Huber et al. 2018). The subfamily Smeringopinae is intermediate in many respects, with most genera confined to dry environments, but with one genus (*Smeringopina*) in tropical African forests (Huber 2013). Ninetinae exemplify the other extreme: they occupy the ground in semi-arid environments that offer a limited number of microhabitats (Huber, Meng, García, et al. 2024; Huber, Meng, Král, et al. 2024). Lastly, in addition to evolutionary potential, interspecific competition may also favor representatives of species-rich groups. For instance, Huber and Acurio (2022) observed that while the introduced species *Modisimus culicinus* (Modisiminae) is becoming more prevalent on Galápagos, the abundance of the native *Galapa bella* (Ninetinae) that occupies the same microhabitat is declining.

In summary, we found that the species-rich subfamilies have higher phenotypic flexibility compared with the species-poor subfamilies, and that trait evolution rate positively correlates with the rates of speciation and microhabitat shift. However, the factors driving the disparity in phenotypic flexibility between species-rich and species-poor groups remain poorly understood. Future research should investigate the roles of genetic and developmental constraints, as well as selective regimes, to better address this knowledge gap. Our study does not directly test microhabitat shifts, as we rely on the evolution of leg proportion measurements as a proxy. Accordingly, further work should also directly evaluate the contributions of habitat and climatic preferences along with biogeographic history.

### The Interplay between Allometry and Evolutionary Flexibility

The allometric scaling relationship between traits is often highly conserved across lineages (Bolstad et al. 2015), and it has been suggested that allometry may limit morphological diversification (Pélabon et al. 2014; Voje et al. 2014). However, empirical evidence shows that the allometric scaling slope and intercept can be modified by natural selection (e.g., Pélabon et al. 2014; Emberts et al. 2023; Chong et al. 2024) and sexual selection (reviewed by Eberhard et al. 2009). Thus, by overcoming the existing constraints in allometric scaling, organisms are able to explore new ecological opportunities (DeLong et al. 2010; Chong et al. 2024).

We found that pholcids generally exhibit a pattern of positive evolutionary allometry between leg length and body size (scaling factor: 1.108) (Fig. 5), indicating that relatively long legs are positively selected. At the subfamily level, however, different subfamilies display distinct allometric slopes and intercepts, suggesting they have undergone varying selection pressures. Interestingly, although Pholcinae, Modisiminae, and Smeringopinae have higher species diversity, speciation rate, trait evolution rate, and phenotypic flexibility, they all showed an allometric pattern similar to isometry, with scaling slopes close to 1 (i.e., 1.012, 1.123, and 0.979) (Fig. 5). One plausible explanation is that stabilizing selection may be acting on the slopes, as suggested by the declining rates of trait evolution revealed in our model-fitting analysis (Supplementary Table S11). This process likely facilitates the specialization of related species into distinct ecological niches (Maher et al. 2022). More specifically, based on the ecological roles of leg length and body size in spiders (Eberhard and Briceño 1984; Eberhard 1992; Huber 1998; Shao et al. 2023), such specializations may be related to locomotion mode, male–male fighting, web construction, courtship, and copulation. For instance, relatively long legs resulting from near-isometry allow spiders to reduce locomotor costs (Pontzer 2007) and bridge relatively large gaps and move efficiently across uneven surfaces (Eberhard 1992), as also predicted by the SGH (Kaspari and Weiser 1999; Pearce-Duvet et al. 2011). The latter adaptation is particularly important, as many Pholcinae, Modisiminae, and some Smeringopinae species inhabit complex forest environments with high levels of three-dimensionality (Huber 2011). Indeed, we observed larger intercepts in the species-rich groups (Pholcinae, Modisiminae, and Smeringopinae) (3.648 ± 0.164, 3.256 ± 0.672, and 4.117 ± 0.208, all with *P*-values << 0.001) compared with the species-poor groups (Arteminae, Ninetinae, and *Artema*, but not *Priscula*) (3.03 ± 0.205, 1.94 ± 0.143, 2.079 ± 0.513, and 4.414 ± 0.399, all with *P*-values << 0.001, except *Artema* with a *P*-value = 0.007) (Fig. 5). This suggests that the species-rich groups generally maintain relatively longer legs than the species-poor groups, as illustrated in Figure 4b and Supplementary Figure S26. On the other hand, how can high phenotypic flexibility in the species-rich groups evolve under stabilizing selection? Stabilizing selection is generally believed to reduce both genetic and phenotypic diversity (Wirthlin et al. 2018; Wolff et al. 2022). We propose three possible explanations. First, isometry and stabilizing selection do not necessarily lead to low phenotypic flexibility. Organisms can still explore a large proportion of morphospace (i.e., larger or smaller trait values) while maintaining slope and intercept unchanged, as exemplified by Pholcinae, Modisiminae, and Smeringopinae (Fig. 4b; Supplementary Figs. S23–S25). Second, environmental heterogeneity favors the maintenance of genetic and morphological variation by favoring divergent adaptive peaks and locally well-adapted phenotypes (Rainey and Travisano 1998; Byers 2005; McDonald and Yeaman 2018; Kalan et al. 2020; Orteu and Jiggins 2020; Boussange and Pellissier 2022). This coincides with the habitat heterogeneity hypothesis, which suggests that increased habitat diversity supports a larger number of species by offering a wider range of niches compared with more homogeneous habitats (MacArthur and MacArthur 1961; Tews et al. 2004; Chen et al. 2020). Lastly, frequent environmental changes or habitat shifts (as revealed by Eberle et al. 2018) can drive a change in optimal trait values, leading to a shift in the adaptive peak and increased rates of trait evolution (Van Tienderen 1991; Coyne et al. 1997; Marques et al. 2022), as well as increased phenotypic variance as suggested by theoretical research (Norberg et al. 2001). For instance, experimental research revealed that greater habitat heterogeneity led to rapid shifts in the feeding behavior of generalist predators including spiders (Staudacher et al. 2018).

On the other side, we found strong positive allometry in the species-poor groups Arteminae, Ninetinae, and *Artema*, with scaling slopes of 1.522, 1.567, and 1.612, respectively, suggesting strong directional selection (Eberhard et al. 2009) on leg length in these groups. *Priscula* had a non-significant negative allometry, suggesting either a decoupling of leg length and body size or an artifact. The steeper slopes in the species-poor groups mean that their leg length increases more rapidly than in the species-rich groups when a change occurs in body size. This suggests that body size has a stronger influence on leg length in the species-poor groups, which could be due to stronger evolutionary constraints. What factors contribute to the low evolutionary flexibility in these species-poor groups? Like stabilizing selection, directional selection also tends to reduce genetic and phenotypic diversity (Assis et al. 2016). Furthermore, these subfamilies typically occupy relatively homogeneous environments, which contribute to their low rates of trait evolution, microhabitat shifts (Fig. 4c,d; Supplementary Fig. S27), speciation, and overall species richness (Fig. 6). With these similar characteristics, Ninetinae exhibits dwarfism whereas *Artema* and *Priscula* show gigantism (Supplementary Fig. S26); both examples of trait specialization may lead to an evolutionary dead end, as indicated by their low net diversification rates and species richness (Fig. 6c). Additionally, trait specialization can constrain long-term diversification by limiting the morphological evolution of other traits, even if the trait is innovative and able to facilitate early radiation (Li et al. 2024).

## Conclusions

Speciation is a complex process, which is influenced by a number of intrinsic and extrinsic factors (Nosil et al. 2009; Rundell and Price 2009; Czekanski-Moir and Rundell 2019). In this study, we discovered that the speciation rate in Pholcidae is positively correlated with the rates of trait evolution, microhabitat shifts, and evolutionary phenotypic flexibility. Our findings indicate that species-rich groups (Pholcinae, Modisiminae, and Smeringopinae) that exhibit allometric scaling close to isometry show the greatest phenotypic flexibility, whereas species-poor groups (Arteminae, Ninetinae, *Artema*, and *Priscula*) with positive allometry are least flexible. Notably, Ninetinae shows a trend toward dwarfism, whereas *Artema* and *Priscula* tend toward gigantism. Species-rich groups are likely influenced by stabilizing selection, whereas species-poor groups experience directional selection; the observed differences in phenotypic flexibility may stem from variations in environmental heterogeneity. Further research is needed to elucidate the causal relationships underlying the correlations observed in this study.

## Supplementary Material

syaf070_Supplemental_Files

## Data Availability

Supplementary materials, data sets, and codes are available from Dryad Digital Repository (https://doi.org/10.5061/dryad.r2280gbnh) as well as the MorphoBank database http://morphobank.org/permalink/?P6008). The raw read data are available at NCBI SRA (BioProject: PRJNA851037).
