# Supplementary material for: The Complex Interplay between Evolutionary Flexibility and Diversification in a Family of Spiders
Source: Syst Biol. 2025 Oct 13;75(5):861–86. doi: 10.1093/sysbio/syaf070 (PMC13445632; doi:10.1093/sysbio/syaf070)
Supplement: Supplementary file 1 [file syaf070_supplemental_files.zip › Supplementary_Data_2.pdf]

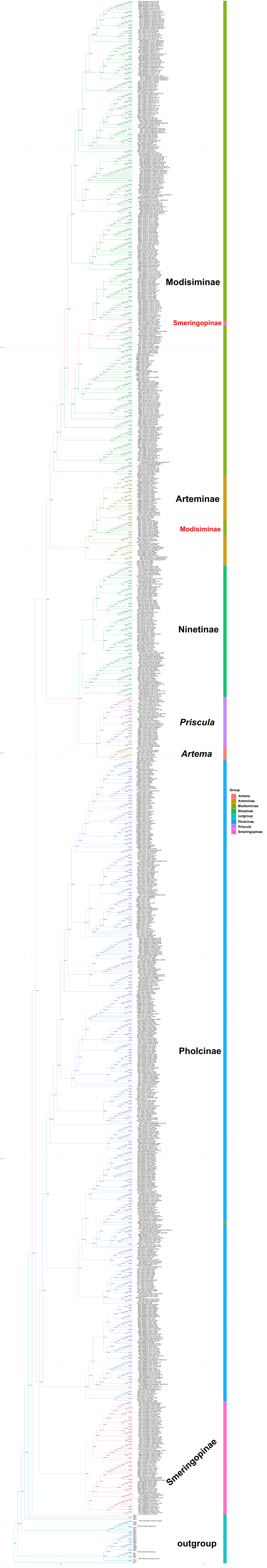

Figure S31. The unpartitioned smart-gap tree based on All UCEs + Sanger data.
The tree was constructed with IQ-TREE, with the optimal model determined by ModelFinder automatically.
The names of wrongly placed taxa are in red. For better visualization, branch length is ignored.
Branch numbers are SH-aLRT support (%) / ultrafast bootstrap support (%)

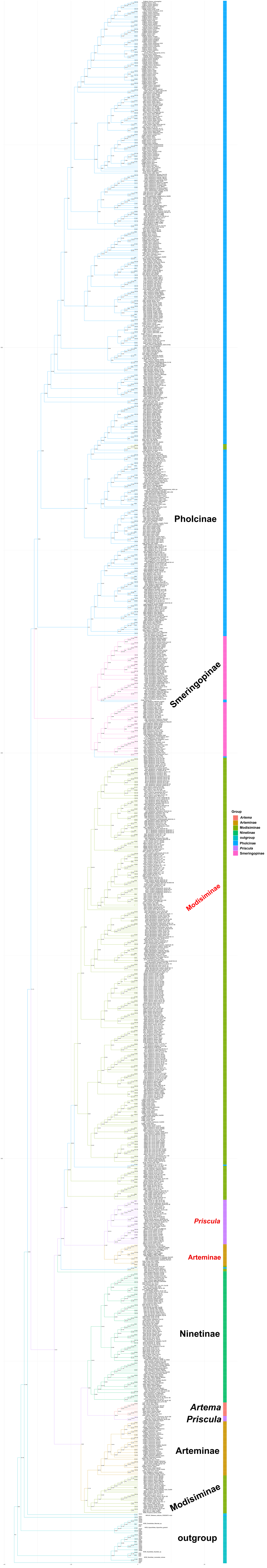

Figure S32. The unpartitioned kpic-smart-gap tree based on All UCEs + Sanger data.
The tree was constructed with IQ-TREE, with the optimal model determined by ModelFinder automatically.
The names of wrongly placed taxa are in red. For better visualization, branch length is ignored.
Branch numbers are SH-aLRT support (%) / ultrafast bootstrap support (%).

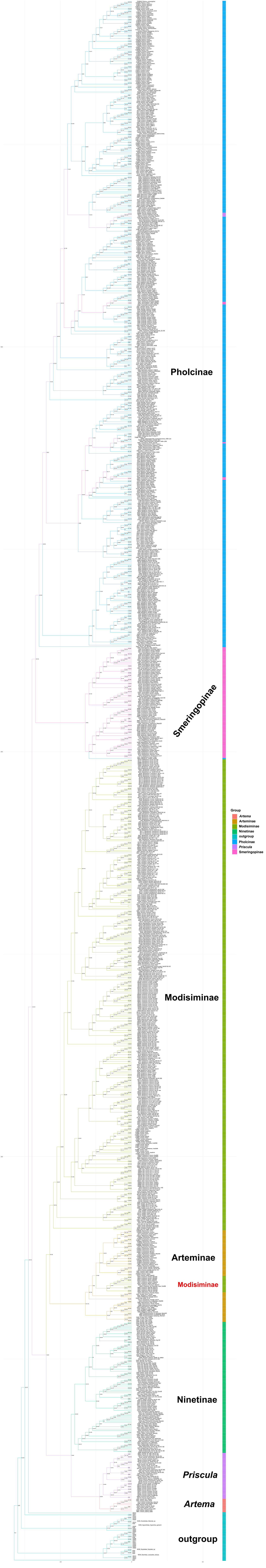

Figure S33. The unpartitioned gappy tree based on All UCEs + Sanger data.
The tree was constructed with IQ-TREE, with the optimal model determined by ModelFinder automatically.
The names of wrongly placed taxa are in red. For better visualization, branch length is ignored.
Branch numbers are SH-aLRT support (%) / ultrafast bootstrap support (%)

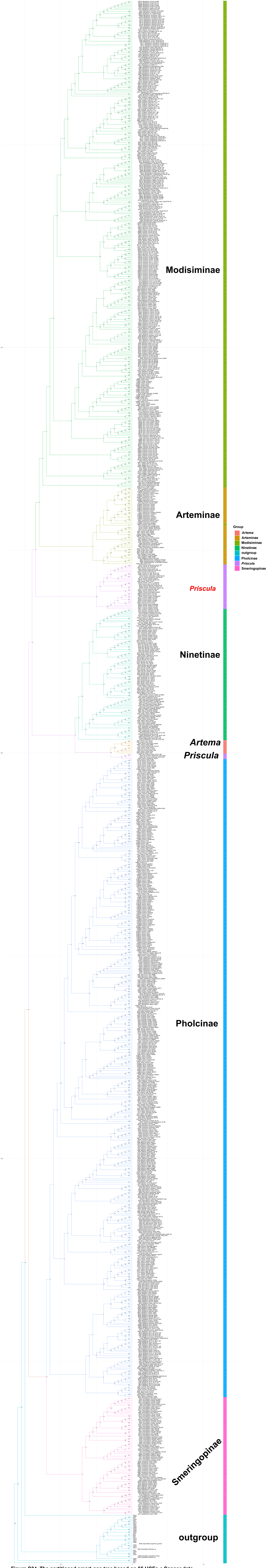

Figure S34. The partitioned smart-gap tree based on 55 UCEs + Sanger data.
The 55 UCEs were selected from matrix 3 and they have the most parsimonious sites.
The tree was constructed with RAxML-NG and the model GTR+R4+I+FO.
The names of wrongly placed taxa are in red. For better visualization, branch length is ignored.
Branch numbers are bootstrap support (%).

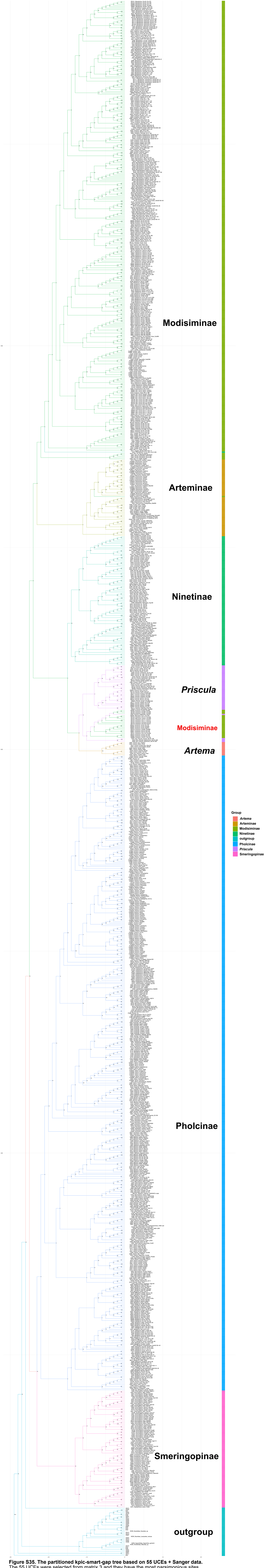

Figure S35. The partitioned kpic-smart-gap tree based on 55 UCEs + Sanger data.
The 55 UCEs were selected from matrix 3 and they have the most parsimonious sites.
The tree was constructed with RAxML-NG and the model GTR+R4+I+FO.
The names of wrongly placed taxa are in red. For better visualization, branch length is ignored.
Branch numbers are bootstrap support (%).

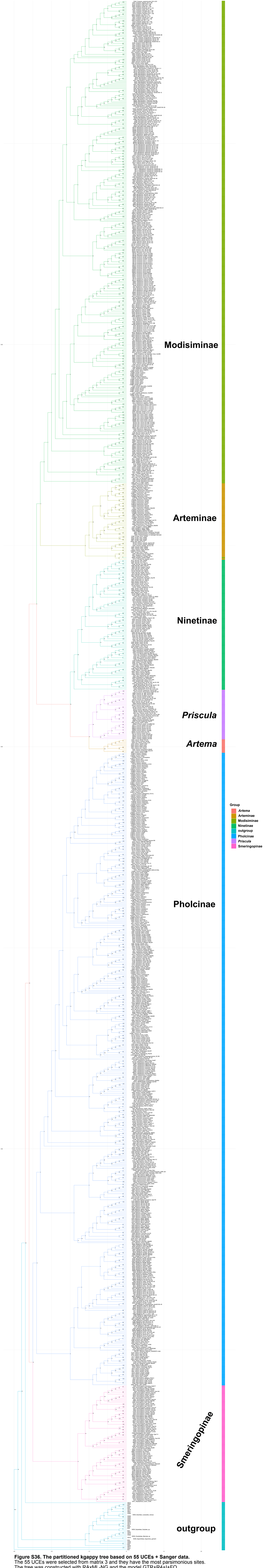

Figure S36. The partitioned kgappy tree based on 55 UCEs + Sanger data.
The 55 UCEs were selected from matrix 3 and they have the most parsimonious sites.
The tree was constructed with RAxML-NG and the model GTR+R4+I+FO.
The names of wrongly placed taxa are in red. For better visualization, branch length is ignored.
Branch numbers are bootstrap support (%).
